# Stent biomechanics in hemodialysis-related left brachiocephalic vein stenosis: from site-specific loading to a staged device-validation framework

**DOI:** 10.3389/fbioe.2026.1961298

**Published:** 2026-09-30

**Authors:** Shanyuan Jin, Chuhan Chen, Guiye Na, Lin Guo, Li Dai, Bing Liu

**Affiliations:** 1 Dalian Medical University, Dalian, China; 2 Department of Orthopedics, The People’s Hospital of Lvshunkou District, Dalian, China; 3 Department of Vascular Surgery, The Second Affiliated Hospital of Dalian Medical University, Dalian, China

**Keywords:** central venous stenosis, computational fluid dynamics, crush resistance, hemodialysis vascular access, in-stent restenosis, left brachiocephalic vein, stent biomechanics, venous stent design

## Abstract

Hemodialysis-related left brachiocephalic vein stenosis is usually managed as one local variant of central venous stenosis, yet its mechanical environment is distinctive. The vein follows a long, multi-axially curved retrosternal course through a narrow window between the manubrium and the aortic arch, bounded by two separate junctions, the lateral venous angle and the medial brachiocephalic–caval confluence. An implanted stent is therefore exposed to focal unidirectional compression, cyclic bending and torsion, short landing zones, and branch-coverage risk, conditions not reproduced by circumferential radial testing. The sternum–aortic corridor reported in affected patients, about 4.6 mm, is narrower than the nominal diameter of stents deployed there, placing the implied compression at or beyond the lowest level characterized in published bench comparisons; pulsatile durability testing covers only cardiac-frequency diametric cycling, and pooled cohorts seldom report the segment separately. This Review synthesizes published anatomical, clinical, engineering, and computational evidence and reframes the problem as a site-specific biomechanical and device-validation question with three validation priority stages (P1–P3). P1 (required) evaluates base-platform mechanics, structure, fatigue, and deployment: focal crush resistance and recovery, radial resistive versus chronic outward force, bending and torsion durability, foreshortening, deployment accuracy, branch preservation and, for covered constructs, covering fatigue. P2 (required) evaluates anatomy-specific hemodynamics of the same platform by patient-specific computational modeling and experimental flow testing, reporting shear, residence, gradient, edge-recirculation, branch-flow, and pressure-loss metrics against matched comparators. P3 (optional) covers an added drug, coating, or surface; baseline thrombogenicity, hemocompatibility, tissue compatibility, and structural integrity remain required base-platform attributes. We state two falsifiable hypotheses with the bench, computational, imaging, and clinical evidence that would support or refute them: H1 (mechanical), that resistance to focal unidirectional crush may govern stent deformation, durability, and in-stent restenosis localization more strongly than circumferential radial resistive force alone; and H2 (hemodynamic), that stent architecture and edge configuration may generate spatially reproducible disturbed-flow regions adding incremental prediction of later in-stent restenosis distribution beyond anatomical and procedural covariates. Direct prospective evidence remains sparse: the framework is a problem definition and validation roadmap, not a clinical algorithm, device ranking, or evidence of superior performance.

## 1 Introduction

Durable vascular access is essential for patients receiving hemodialysis (HD). Central venous stenosis (CVS) or occlusion (CVO) can cause access dysfunction, venous hypertension, inadequate dialysis, arm or cervicofacial swelling, chest-wall collateral veins, elevated venous dialysis pressure, prolonged post-puncture bleeding, abnormal access flow, and recurrent access thrombosis (Echefu et al., 2023). The brachiocephalic vein is among the segments most frequently involved in HD-related central venous disease (Andrawos et al., 2021). In a single-center prospective study of 152 maintenance HD patients evaluated by computed tomography venography (CTV), 20 (13.1%) had CVS, the brachiocephalic vein was the most frequently involved segment (65% of those 20), and 6-month primary patency after intervention was 44.4% (Singh et al., 2025). These proportions rest on small denominators in one cohort. Left brachiocephalic vein (LBV) stenosis or occlusion may also cause internal jugular venous reflux, headache, nausea, orbital venous congestion, and, rarely, intracranial hemorrhage (Xiong and Zhang, 2024; Sasaki et al., 2023).

The LBV differs substantially from the right brachiocephalic vein: it crosses the midline before entering the superior vena cava (SVC), follows a long retrosternal course, and lies between the sternum and the aortic arch/brachiocephalic artery (Vertemati et al., 2020; White and Deeb, 2023). Three-dimensional studies demonstrate multi-axial angulation, marked interindividual variability, and associations among a shortened sternum–LBV distance, a left-sided venous route, and CVS risk (Vertemati et al., 2020). Dialysis cohorts further show that LBV stenosis can arise without prior central venous catheterization, supporting extrinsic compression as an important anatomical driver, particularly in patients lacking a catheter history or with imaging evidence of compression (Itkin et al., 2004; Wan et al., 2024).

Previous reviews and guidance have primarily treated central venous stenosis as a broad vascular-access problem, emphasizing pathophysiology, clinical presentation, indications for intervention, and access-preservation strategies across central venous segments (MacRae et al., 2005; Echefu et al., 2023; Agarwal, 2015; Lok et al., 2020). These works remain essential for clinical management, but they do not treat the LBV as a distinct retrosternal, junction-adjacent, mechanically compressed design environment, and to our knowledge no review has addressed it as a site-specific design target for venous stents in hemodialysis.

Imaging-detected LBV stenosis, however, is not synonymous with disease requiring intervention. Collateral pathways may preserve venous return, and asymptomatic CVS should generally not be treated prophylactically (Agarwal, 2015; Lok et al., 2020). Kidney Disease Outcomes Quality Initiative (KDOQI) guidance and related summaries emphasize that a decision to treat an access lesion should be based on both clinical indicators and imaging evidence, rather than stenosis percentage alone (Lok et al., 2020; Lok and Rajan, 2022). This threshold matters because unnecessary intervention may injure the endothelium, accelerate restenosis, and consume future access options (Agarwal, 2015; Lok et al., 2020).

Symptomatic CVS/CVO is treated mainly with percutaneous transluminal angioplasty (PTA), bare-metal stents (BMS), and stent grafts (SG) (Andrawos et al., 2021; Agarwal, 2015). PTA is simple and repeatable but is limited by elastic recoil and early recurrence (Agarwal, 2015). BMS provide radial support but do not prevent tissue ingrowth or neointimal hyperplasia (Liu et al., 2024; Yu et al., 2024). SG reduce tissue ingrowth through a physical barrier, yet edge stenosis, branch-vein coverage, thrombosis, and repeated reintervention remain important limitations (Lok et al., 2020; Liu et al., 2024; Anaya-Ayala et al., 2011). Current interventions can restore outflow in selected patients, but durable access preservation often depends on surveillance and repeat procedures (Andrawos et al., 2021; Chawla et al., 2025). Table 1 summarizes the main failure boundaries of these options.

**TABLE 1:** Current endovascular options as failure-boundary evidence for HD-related LBV/CVS. This table compares PTA, BMS, and SG by mechanism, advantages, limitations, and device-validation implications. Abbreviations: HD, hemodialysis; LBV, left brachiocephalic vein; CVS, central venous stenosis; PTA, percutaneous transluminal angioplasty; BMS, bare-metal stent; SG, stent graft; ePTFE, expanded polytetrafluoroethylene.

Treatment	Main features/mechanism	Advantages	Limitations	Implications for LBV/CVS
PTA	Balloon dilation restores the stenotic lumen and remains the foundational first-line treatment for symptomatic central venous stenosis (Andrawos et al., 2021; Agarwal, 2015; Lok et al., 2020; Wu et al., 2020)	Relatively simple, minimally invasive, and repeatable; does not permanently occupy venous branches or future access resources	Elastic recoil and early recurrence are common in central veins; durability is limited for LBV lesions with marked extrinsic compression, often requiring repeated reintervention (Agarwal, 2015; Wu et al., 2020; Wan et al., 2025)	Defines the initial and diagnostic treatment boundary. Residual stenosis, marked recoil, or early symptomatic recurrence highlight scenarios in which additional support has been explored rather than proving a universal stenting pathway
Bare-metal stent (BMS)	Provides sustained radial support to overcome post-PTA recoil and maintain luminal patency (Liu et al., 2024; Yu et al., 2024; Chawla et al., 2025; Haage et al., 1999)	More resistant to short-term recoil than PTA; deployment is mature and may be used in selected recurrent or recoil-prone lesions	Metallic struts do not prevent tissue ingrowth; neointimal hyperplasia and restenosis are prominent; long-term patency benefit is limited and deformation or fracture may occur (Yu et al., 2024; Chawla et al., 2025; Hammer et al., 2000; Quaretti et al., 2016)	Defines the limitation of radial support alone in compressed and multi-axial LBV anatomy. Its main relevance to this review is mechanical support without inherent biological antiproliferative function
Stent graft (SG)	A metal scaffold covered with ePTFE or similar material forms a physical barrier to reduce tissue ingrowth through struts (Lok et al., 2020; Liu et al., 2024; Anaya-Ayala et al., 2011; Wan et al., 2025; Quaretti et al., 2016)	Available evidence provides comparatively stronger support for short- to mid-term patency; SG may be advantageous in selected LBV lesions with marked recoil, recurrence, or extrinsic compression	Edge stenosis, intrastent thrombosis/thrombus organization, and reintervention still occur; covering the internal jugular or subclavian ostium may permanently consume important vascular resources (Lok et al., 2020; Wan et al., 2025; Ginsburg et al., 2015)	Provides boundary evidence for recoil-prone or recurrent symptomatic lesions, while emphasizing the need to validate landing-zone planning, edge behavior, and branch preservation before generalizing SG use in the LBV.

Against this background, we frame HD-related LBV stenosis as a site-specific bioengineering and venous-device validation problem rather than a local subtype of generic CVS, with emphasis on anatomy-driven failure modes and validation requirements for LBV-oriented stent concepts. Uncovered nitinol scaffolds, covered stent grafts, and surface-functionalized concepts are treated as distinct biomaterial-device families, each with its own endpoints: mechanical survival in a retrosternal compression window, branch-safe and low-foreshortening deployment, flow compatibility at the stent body and edges, covering or coating durability, hemocompatibility, and access preservation. The analysis centers on the loading environment and the validation endpoints it demands.

Two review-derived, falsifiable hypotheses organize the argument. H1 (mechanical) concerns focal unidirectional crush within the sternum–aortic window as a determinant of stent deformation, durability, and in-stent restenosis localization. H2 (hemodynamic) concerns stent architecture and edge configuration as generators of reproducible disturbed flow that predicts later restenosis distribution. Both are stated in full, with supporting evidence and refutation conditions, in the section on biomaterial and venous-device constraints.

## 2 Methods: review approach and evidence framework

This article is a structured critical narrative Review with a translational focus on functional biomaterials and venous-device design. Because direct LBV evidence is limited and the relevant literature spans anatomy, dialysis access, intervention, venous stent pathology, hemodynamics, biomaterials, surface engineering, and device mechanics, our aim was to integrate evidence across disciplines and identify testable design and validation priorities. It is not a systematic or scoping review, meta-analysis, or guideline; no quantitative synthesis, formal risk-of-bias grading, treatment recommendation, or device ranking was attempted. Two questions organize the argument: why the LBV should be considered separately from generic CVS, and which mechanical, hemodynamic, deployment, and biomaterial dimensions warrant future validation. The hypotheses and staged framework organize the published evidence and define questions for future testing; they are not based on new experimental or computational results.

Literature was identified through targeted searches and citation tracking. PubMed/MEDLINE was the principal biomedical database, Web of Science and Embase broadened coverage of the clinical, engineering, and device literature, and Google Scholar was used selectively for cited-reference and device searches. Searches were updated through 20 May 2026 and organized around four domains: direct LBV/brachiocephalic clinical and anatomical evidence; general HD-related CVS evidence and guidance; in-stent restenosis (ISR), hemodynamic, and mechanobiological evidence; and venous-stent engineering evidence. Targeted citation surveillance through 20 July 2026 identified later studies affecting the interpretation of H2 and the P2 comparator logic. Database-adapted search strings and evidence-use boundaries are given in Supplementary Table S2; because the aim was interpretive synthesis rather than exhaustive retrieval, no PRISMA-style selection flow is reported. Generative AI assisted with language and structural editing and with schematic drafts after the evidence base had been assembled; it did not select sources or assign evidence-proximity labels, and all outputs were verified by the authors (see Acknowledgments).

### 2.1 Source selection and critical appraisal

The searches identified sources relevant to the Review’s central arguments and were not designed to estimate intervention effects. Sources were selected for conceptual relevance, anatomical proximity to the LBV, methodological informativeness, and their capacity to support a clinical, mechanistic, or engineering inference. Citation chaining from key guidelines, reviews, LBV studies, and device-engineering papers identified additional sources. Selection and interpretation were iterative, consistent with a critical narrative Review.

Sources considered included clinical studies, case series, reviews, meta-analyses, and guidelines on LBV, brachiocephalic, subclavian, or SVC disease in HD or advanced chronic kidney disease, together with interventional studies reporting technical success, patency, reintervention, or access preservation. Mechanistic studies of venous ISR, thrombus organization, neointimal hyperplasia, or shear-related endothelial responses and engineering studies of deployment, foreshortening, flexibility, crush resistance, covering, drug delivery, or surface modification were also included.

Sources confined to peripheral arterial, coronary, or lower-extremity venous disease without interpretable relevance to central veins, lacking lesion localization or interpretable outcomes, or available only as conference abstracts were generally excluded. Pediatric, non-dialysis catheter, and iliofemoral venous stent studies were used only as indirect evidence on device thrombosis (Fu et al., 2024), stent mechanics, or venous stent failure. Full criteria are given in Supplementary Table S2.

### 2.2 Structured evidence mapping and interpretive hierarchy

Evidence proximity was assigned to individual claims rather than to whole papers, so a single source may support different claims at different proximity levels: an LBV imaging study, for example, may directly support an anatomical observation yet only indirectly motivate a device-test requirement. Four labels defined in Table 2 are used throughout: Direct LBV, Adjacent HD-CVS, Analogous engineering, and Mechanistic hypothesis. They indicate anatomical and experimental proximity to the specific inference, not study quality or formal certainty. A site-specific factual assertion requires Direct LBV evidence; the remaining layers may constrain interpretation or generate a testable hypothesis, but cannot by themselves establish LBV causality, device efficacy, or an acceptance threshold. Where a claim draws on more than one layer, the nearest supporting evidence and the residual LBV-specific validation gap are stated explicitly.

**TABLE 2:** Evidence-proximity framework and interpretive boundaries used in this Review. Evidence proximity is assigned to individual claims and indicates anatomical or experimental closeness to the specific LBV inference, not methodological quality or certainty.

Evidence layer	Representative source types	Appropriate inference	Not supported	Role in this review
Direct LBV	LBV/left innominate anatomy, imaging, lesion-localized clinical series, and LBV case reports	Site-specific anatomy, compression phenotype, presentation, and associations observed in the LBV.	Causal dominance, comparative device efficacy, or universal thresholds from small or non-comparative studies	Defines the anatomical boundary condition and anchors LBV-specific claims
Adjacent HD-CVS	HD-related central venous guidance, reviews, and mixed brachiocephalic/subclavian/SVC cohorts without separable LBV data	General indications, access-preservation principles, recurrent failure phenotypes, and treatment boundaries	LBV-specific effect sizes, patency estimates, or proof that one mechanism dominates in the LBV.	Constrains clinical interpretation where direct LBV data are sparse
Analogous engineering	Venous-stent bench studies; SVC/iliofemoral deployment; AVF/AVG or bifurcation CFD/FSI; nitinol, fatigue, and covering studies	Candidate loading modes, metrics, test methods, and design trade-offs requiring LBV adaptation	LBV device performance, acceptance thresholds, or superiority of a commercial design	Generates candidate P1/P2 validation requirements
Mechanistic hypothesis	Cellular, animal, and non-LBV vascular studies of shear signaling, inflammation, thrombosis, and remodeling	Biological plausibility, candidate pathways, and exploratory biomarkers	A causal LBV ISR pathway or therapeutic efficacy without LBV tissue or model validation	Defines exploratory P3 questions; does not drive device recommendations

Critical appraisal was qualitative and tailored to source type rather than based on formal GRADE or risk-of-bias instruments. Clinical cohorts were appraised with attention to lesion segment, catheter history, patency definition, follow-up, and reintervention triggers. Case reports were used only as evidence of a rare phenotype or technical feasibility. Bench, computational, cellular, and animal studies were assessed according to their relevance to LBV compression, angulation, and high-flow access conditions. Direct LBV studies were prioritized for site-specific conclusions.

## 3 Anatomy-specific problem: triple-hit origins of LBV device failure

Brachiocephalic vein stenosis is multifactorial, but the LBV is not simply a smaller clinical subtype of generic central venous stenosis. It constitutes an anatomy-specific venous-device environment in which a long retrosternal course, multi-axial angulation, sternum–aortic compression, catheter/device contact, and access-related high-flow exposure may converge on the same implanted segment. Figure 1 gives a conceptual, hypothesis-generating overview of the interacting anatomical, mechanical, hemodynamic, thrombotic, inflammatory, and proliferative processes involved. The proposed triple-hit model organizes three candidate exposure domains, which Figure 2 maps to claim-specific test paths: anatomical vulnerability may predispose the vein to extrinsic compression; catheter- or device-related endothelial injury may contribute to local inflammatory or proliferative responses; and high-flow exposure may coexist with regionally disturbed flow and amplify abnormal remodeling. This classification translates anatomy and pathophysiology into device-failure phenotypes and candidate validation needs without implying temporal or causal necessity, complementing conventional pathological outcome categories (Echefu et al., 2023).

**FIGURE 1:**
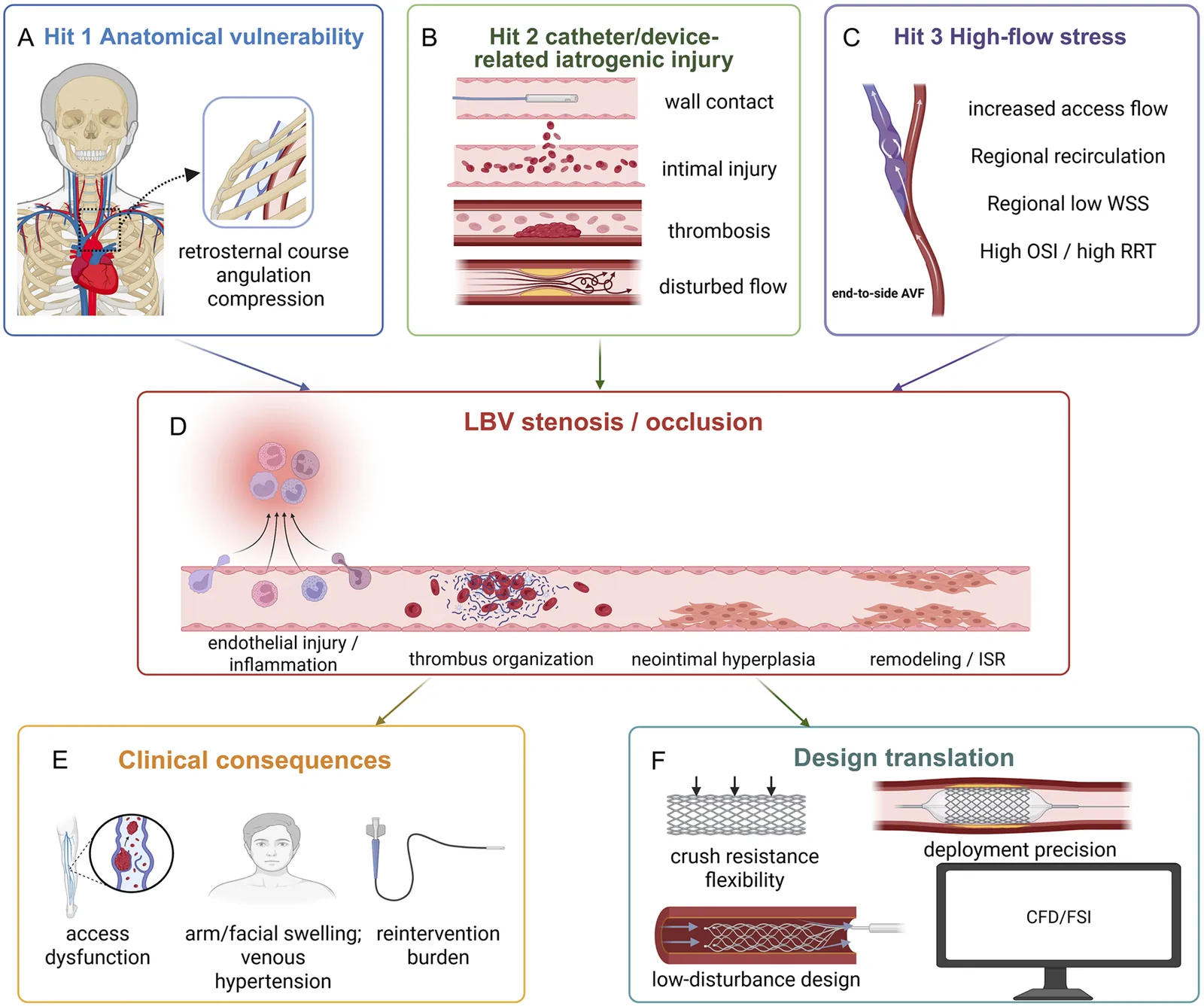
Conceptual overview of interacting anatomical, mechanical, hemodynamic, thrombotic, and proliferative processes in hemodialysis-related left brachiocephalic vein stenosis and post-intervention failure. **(A)** Hit 1, anatomical vulnerability: the long transverse retrosternal course of the left brachiocephalic vein (LBV), its multi-axial angulation, and focal compression between the sternum or manubrium and the aortic arch. **(B)** Hit 2, catheter- or device-related iatrogenic injury: wall contact, intimal injury, thrombosis, and locally disturbed flow. **(C)** Hit 3, high-flow exposure after creation of an end-to-side arteriovenous fistula (AVF), with increased access flow, regional recirculation, low WSS, high OSI, and high RRT. **(D)** Candidate interacting remodeling processes at the site of LBV stenosis or occlusion, from endothelial injury and inflammation through thrombus organization and neointimal hyperplasia to remodeling and in-stent restenosis (ISR). **(E)** Clinical consequences, including access dysfunction, arm or facial swelling and venous hypertension, and reintervention burden. **(F)** Translation of these exposures into device-design and validation targets: crush resistance and flexibility, deployment precision, low-disturbance design, and computational fluid dynamics or fluid–structure interaction assessment. Although access flow may increase bulk velocity and wall shear in some regions, curvature, compression, venous junctions, stent edges, and struts may produce localized flow separation, recirculation, low or oscillatory wall shear stress, and prolonged blood residence. The processes shown are interacting and non-exclusive and may occur independently, in parallel, or with reciprocal interaction before converging on lumen re-narrowing, in-stent restenosis, thrombosis, patency loss, or reintervention; their graphical arrangement does not represent an obligatory temporal sequence. Arrows indicate proposed, testable relationships rather than established LBV-specific causality, device efficacy, or a clinical treatment pathway. Abbreviations: AVF, arteriovenous fistula; ISR, in-stent restenosis; LBV, left brachiocephalic vein; OSI, oscillatory shear index; RRT, relative residence time; WSS, wall shear stress. Created in BioRender. Jin S. (2026) https://BioRender.com/tc2h4q1.

**FIGURE 2:**
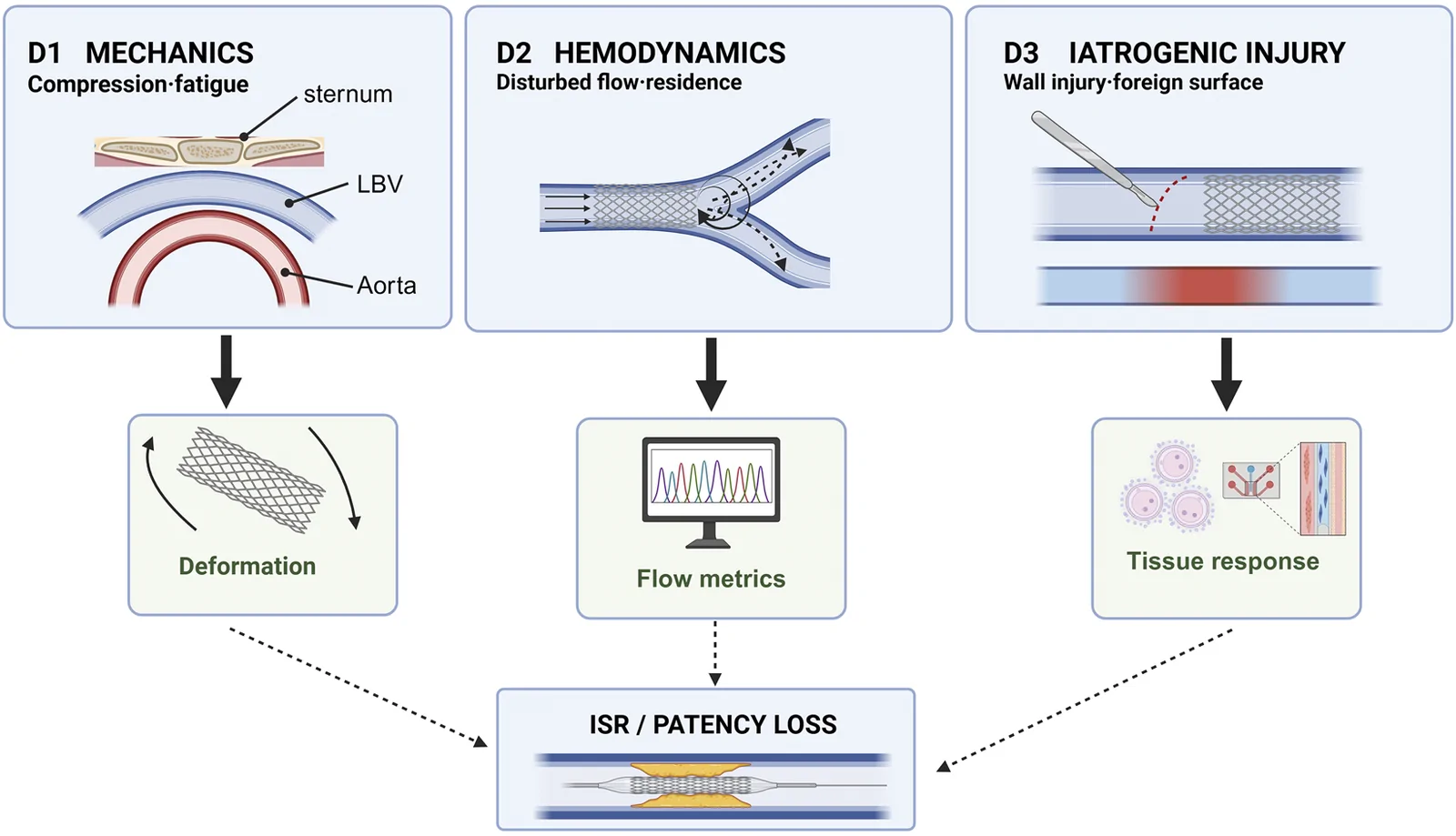
Triple-hit framework and corresponding testable readouts for failure of the stented left brachiocephalic vein. The three exposure domains are labeled D1–D3 so that they remain distinct from the two falsifiable hypotheses H1 and H2 stated in the text. D1 is anatomy-related mechanical loading, including focal external compression and cyclic deformation or fatigue. D2 is anatomy- and stent-related hemodynamic disturbance, including flow separation, recirculation, and prolonged blood residence. D3 is iatrogenic wall injury and foreign-surface exposure associated with prior catheter or device contact and subsequent implantation. Hypothesis H1 formalizes the mechanical exposure represented by D1, and hypothesis H2 formalizes the disturbed-flow environment on which D2 and D3 converge. Each domain is linked to a corresponding group of measurable mechanical, hemodynamic, or tissue-response readouts. The three domains may coexist and interact in contributing to in-stent restenosis and patency loss. Solid arrows indicate domain-to-readout mapping, whereas dashed arrows indicate proposed associations with clinical outcomes rather than established causality or an obligatory temporal sequence. Abbreviations: ISR, in-stent restenosis; LBV, left brachiocephalic vein. Created in BioRender. Jin S. (2026) https://BioRender.com/r201pok.

### 3.1 Anatomical segments and phenotype definitions used in this review

In this Review, the LBV course is divided into three working segments, consistent with three-dimensional reconstructions of its anatomy (Salik et al., 2007; Vertemati et al., 2020) and with segment-based clinical practice for stent-type selection (Tabriz and Arslan, 2022). The lateral segment begins at the lateral venous angle, where the left internal jugular and left subclavian veins form the LBV, and extends toward the sternum–aortic arch corridor. The retrosternal compression segment lies beneath the manubrium and anterior to the aortic arch, and its sternum–aortic crush window anchors H1. The medial segment terminates at the brachiocephalic–SVC confluence. The lateral venous angle and the medial brachiocephalic–SVC confluence are anatomically distinct junctions and should not be collapsed into a single combined junction. This descriptive framework keeps anatomical, landing-zone, and branch-preservation claims tied to the specific portion of the vein concerned.

The evidence reviewed below can also be organized into overlapping failure phenotypes reflecting the dominant driver in a given study rather than mutually exclusive categories. An extrinsic-compression-dominant phenotype is characterized by sternum–aortic narrowing without antecedent catheterization (Itkin et al., 2004; Oguzkurt et al., 2005; Wan et al., 2024). A catheter/iatrogenic-dominant phenotype follows tunneled catheter placement, particularly by a left-sided route, for which clinical and anatomical studies support route-related risk and unfavorable angulation without quantifying catheter–wall contact pressure (Salgado et al., 2004; Salik et al., 2007; Yevzlin, 2008). A proposed high-flow-amplified phenotype follows arteriovenous access creation, but its LBV-specific causal evidence remains indirect, drawn from arteriovenous fistula (AVF) and graft (AVG) hemodynamics and observations of previously silent central venous disease (He et al., 2025; Ninno et al., 2025; Renaud et al., 2012; Yamaguchi et al., 2020). A thrombus-organization/ISR-dominant phenotype presents as recurrent in-stent restenosis after an initially successful intervention (Yu et al., 2024; Chawla et al., 2025). Most cases are probably mixed.

Two further distinctions recur throughout the Review. First, imaging-detected narrowing is distinguished from clinically significant stenosis requiring intervention, consistent with current guidance (Lok et al., 2020; Lok and Rajan, 2022); Table 3 translates this distinction into device-constraint strata. Second, the left–right distinction that motivates this Review rests on convergent anatomical evidence rather than a single finding. The evidence includes a longer and more angulated course (Vertemati et al., 2020), a sternum–aortic crossing that the right brachiocephalic vein does not share (White and Deeb, 2023), and a smaller baseline left internal jugular caliber in roughly one-third of patients (Agarwal, 2015). No comparably specific device-design literature exists for the right brachiocephalic vein; this convergence is the evidentiary basis for treating the LBV as a distinct design target rather than a representative example of brachiocephalic stenosis.

**TABLE 3:** Anatomical–clinical contexts translated into device-validation constraints for HD-related LBV/CVS. Each row identifies the failure phenotype a context reveals and the resulting device-design constraint; the table is a constraint-mapping tool, not a treatment pathway or product recommendation. In this table, “stent/device translation” may involve SG, dedicated venous stents, or selected salvage BMS use, depending on lesion segment, landing-zone anatomy, branch-coverage risk, device availability, and local expertise; because LBV-specific randomized trials are lacking, it standardizes narrative interpretation and extracts device-design constraints rather than recommending any device or pathway.

Anatomical-clinical context (boundary condition)	Key assessment	Failure phenotype revealed (boundary evidence)	Device-constraint translation	Cautions and evidence boundaries
All suspected LBV/CVS patients	Whether clinical indicators coexist with imaging/angiographic stenosis: arm or cervicofacial swelling, venous hypertension, inadequate dialysis flow, prolonged post-puncture bleeding, access dysfunction, etc.	Imaging-only stenosis without symptoms or access dysfunction is used as the boundary condition for non-intervention in current guidance; baseline imaging, access flow, symptoms, and follow-up define the clinical context	Routine PTA or stenting for asymptomatic imaging-only stenosis is not used as device-efficacy evidence; the following strata become relevant only when clinical indicators are present	This threshold reduces overtreatment and protects future access resources. Device studies should not define success by stenosis percentage alone
No catheter history and marked extrinsic compression	CTV or venography shows sternum–aortic compression, long transverse LBV course, or multi-axial angulation; PTA-related recoil, residual stenosis, or early recurrence risk is high	PTA is represented as the initial luminal-opening and diagnostic boundary in available studies; residual stenosis >30%, marked recoil, or early symptomatic recurrence identify support-dependent failure phenotypes	SG or dedicated venous stent concepts in this stratum translate into requirements for crush resistance, flexibility, and deployment precision; BMS use is interpreted as selected salvage/boundary evidence	Landing-zone and branch-coverage risks define major validation boundaries. Early support in this stratum remains a strictly selected, hypothesis-generating scenario requiring prospective validation
Prior catheter/device-related intimal lesion	History of ipsilateral central venous catheter, PICC, port, or repeated catheterization; lesions may be dominated by endothelial injury, intimal hyperplasia, thrombus organization, and multifocal stenoses	PTA and repeat intervention data define the baseline failure pattern: dilation, surveillance, and reintervention are often needed to maintain access function	SG evidence for recurrence, recoil, or failure of PTA to maintain patency defines the need for edge-control, tissue-ingrowth, and thrombus-organization endpoints; BMS has limited long-term advantage as a routine design solution	The entire central venous system and prior device factors frame the interpretation. Pharmacologic therapy cannot substitute for validation of mechanical restenosis control
Occlusion, long-segment disease, or failure of conventional guidewire crossing	CVO, long-segment occlusion, recurrent re-occlusion, or failed conventional guidewire crossing, often with collateral circulation and complex reintervention history	Complex CVO evidence is treated as high-risk boundary evidence from experienced centers rather than as a general device pathway	Successful sharp recanalization often creates a need to study post-recanalization support, reintervention feasibility, and alternatives such as HeRO graft, right atrial bypass, lower-limb access, or other salvage options	Sharp recanalization has higher adverse-event risk and is not generalized to ordinary stenosis. Alternative access evidence applies only to highly selected end-stage access patients
High-risk junction/branch involvement	Lesion is near the lateral venous angle (left internal jugular–subclavian junction) or the medial brachiocephalic–SVC confluence; stent or SG placement may cover distinct inflows and affect future catheter/access planning	Three-dimensional imaging and landing-zone planning define the boundary condition; durable symptom control without permanent branch coverage illustrates the value of vascular-resource preservation	If a stent concept is tested in this anatomy, key constraints include low foreshortening, precise length control, branch access, and potential short-segment, bifurcation/Y-shaped, fenestrated, or customized strategies	Important-branch coverage carries vascular-resource cost. These scenarios highlight the need for multidisciplinary interpretation and branch-preservation endpoints
Cardiac implantable electronic device lead-related CVS	Lesion is related to pacemaker/defibrillator leads, or the stent path crosses existing leads; risks include lead entrapment, infection, and future device-management difficulty	Multidisciplinary assessment defines the boundary condition because lead removal, revision, conversion, infection risk, and access planning influence interpretation	PTA data may represent short-term relief or bridging evidence; stent evaluation across or near leads must account for lead entrapment, infection, and future device-management constraints	Device conflict risk is high. The relevant design endpoints shift from single-session opening to long-term device safety, access preservation, and reintervention feasibility

### 3.2 First hit: anatomical vulnerability and extrinsic compression

The asymmetric embryological and adult configuration of the left and right brachiocephalic veins provides a congenital basis for treating the LBV as a high-risk site of CVS.

#### 3.2.1 Side-specific differences in macroscopic anatomy

Unlike the shorter and more vertically oriented right brachiocephalic vein, the LBV crosses the upper mediastinum from the left side, travels behind the manubrium and anterior to the aortic arch and its branches, and drains into the SVC along a long, tortuous retrosternal course with several principal angulations (Vertemati et al., 2020; White and Deeb, 2023). This sternum–aortic arch corridor exposes the vein to chronic osteoarterial extrinsic compression, and reports of crushed stents in benign LBV stenosis among HD patients further suggest persistent mechanical loading at this site (Hammer et al., 2000). Figure 3A shows the sternum–aortic corridor, whereas Figure 3B contrasts circumferential radial loading with focal anterior–posterior crush.

**FIGURE 3:**
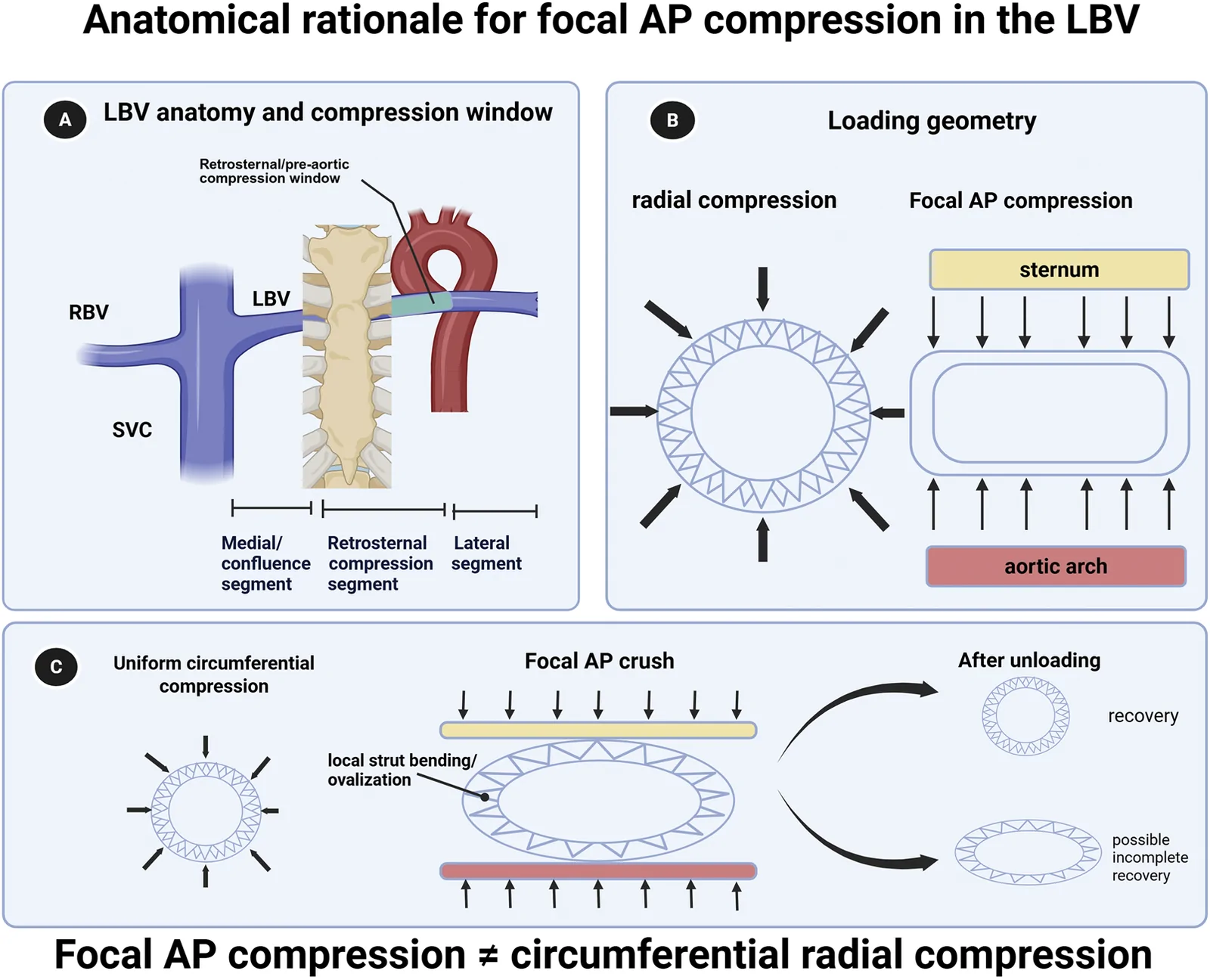
LBV anatomy and the distinction between radial support and focal crush. **(A)** An anterior-view schematic shows the long transverse LBV course and three working segments: the medial/confluence segment at the brachiocephalic–SVC junction, the retrosternal compression segment containing the sternum–aortic window, and the lateral segment extending toward the venous angle. The left internal jugular and left subclavian tributaries that form the lateral venous angle are omitted from this simplified drawing. **(B)** Circumferential radial testing is distinguished from focal anterior–posterior crush testing. **(C)** Schematic stent responses under uniform circumferential compression and focal crush are contrasted, including local strut bending or ovalization and recovery or possible incomplete recovery after unloading; this panel does not present experimental data. RRF/COF and crush resistance describe different loading geometries and should not be treated as interchangeable; LBV-oriented testing should combine focal compression with patient-relevant curvature, end constraints, fatigue assessment, and junction-specific branch preservation. The schematics are not to scale. Abbreviations: AP, anterior–posterior; COF, chronic outward force; LBV, left brachiocephalic vein; RBV, right brachiocephalic vein; RRF, radial resistive force; SVC, superior vena cava. Created in BioRender. Jin S. (2026) https://BioRender.com/e3uxgua.

#### 3.2.2 Quantification of three-dimensional angulation and anatomical risk factors

Vertemati et al. used three-dimensional reconstruction to characterize the multi-axial angulation and long, curved retrosternal course of the LBV (Vertemati et al., 2020). Consistent with an anatomical-compression mechanism, Shi et al. compared 9 hemodialysis patients with left innominate vein stenosis and 10 without and found a markedly narrower sternum–aortic space in the stenosis group (4.55 ± 2.67 mm versus 15.25 ± 6.12 mm; *p* < 0.001), with a correspondingly smaller vein diameter (1.69 ± 1.55 mm versus 8.71 ± 2.33 mm) (Shi et al., 2014). This cohort is very small, and its cross-sectional design cannot separate cause from consequence, since a vein narrowed for other reasons will also measure a smaller residual diameter within the same corridor. Supporting a structural rather than purely dialysis-related basis, a retrospective CT-angiography series of 212 asymptomatic non-dialysis adults found left innominate vein narrowing (>25% area loss at the crossing point) in 35.4% of cases, with a smaller aortic arch-to-sternum space and a more proximal crossing site as independent correlates (Guo et al., 2017). Compression geometry therefore appears to exist in the general adult population and to be more prevalent, and more often symptomatic, once access-related high flow is superimposed. The quantitative relationship between corridor width, residual lumen, and device-relevant loading remains unestablished.

These measurements have a device-relevant reading. For a stent of 12 mm nominal diameter, within the range used in thoracic central venous practice, constraint within a 4.6 mm corridor corresponds to roughly 38% of nominal diameter, and to about 60% even one standard deviation above that corridor mean. Published venous-stent bench comparisons, including the dataset that anchors H1 below, characterize devices only down to 50% of nominal diameter (Dabir et al., 2018), so the implied compression lies at or beyond the lowest level at which these devices have been described. Three caveats bound the estimate. The corridor was measured on static computed tomography in a 9-versus-10-patient cohort and without an implanted device. A deployed stent displaces mediastinal soft tissue, so the *in situ* corridor is not rigid. Corridor width and residual lumen are not independent measurements in an already narrowed vein. The calculation therefore sizes the plausible compression range rather than defining a design threshold, and identifies the missing quantity: the width of the sternum–aortic contact footprint, which sets the loaded arc in the focal loading problem below.

This baseline caliber difference may interact with the tortuous left-sided pathway after catheter placement or fistula creation, increasing susceptibility to mechanical stress injury. The caliber observation itself is Adjacent HD-CVS evidence, and the proposed mechanical interaction is a hypothesis rather than a measured effect.

#### 3.2.3 Extrinsic compression is a common site-specific driver in LBV stenosis

Wan et al. studied 84 HD patients with LBV stenosis or occlusion, of whom 62 (73.8%) had no history of central venous catheterization; this proportion describes catheter history, not the share of lesions attributable to extrinsic compression. Patients without prior catheterization more often had stenosis rather than occlusion, a narrower sternum–aortic interval, and higher rates of balloon-related elastic recoil and stent placement (all *p* < 0.05) (Wan et al., 2024). Itkin et al. reviewed 106 left upper-extremity fistulograms from 48 HD patients and observed some degree of extrinsic compression of the left innominate vein in 21 of 48 patients (44%), graded as mild in 25%, moderate in 13%, and severe in only 6%, so the 44% figure denotes any detectable compression rather than a high-grade lesion (Itkin et al., 2004). Among patients with anatomical compression, only 19% had a prior left-sided subclavian or internal jugular catheterization, whereas 82% of those with intrinsic stenosis did (Itkin et al., 2004). Extrinsic compression therefore appears common, and may predominate, in LBV subgroups without catheter history or with clear imaging evidence of compression. Because catheter history, lesion definitions, compression grading, and imaging criteria differ across cohorts, these data do not establish extrinsic compression as the universal cause of LBV stenosis.

Other clinical observations support this interpretation. In a cohort of dialysis patients treated with PTA for central venous disease, in which LBV involvement was the most common finding, primary patency was 81%, 73%, 65%, and 57% at 3, 6, 9, and 12 months (Banshodani et al., 2014). A case of mediastinal cystic-mass compression suggests that creating a left-sided access may rapidly unmask a pre-existing outflow bottleneck (Yamaguchi et al., 2020). One clinically testable hypothesis is that, if compression geometry is an anatomical baseline unmasked by high-flow access, cross-sectional imaging of the corridor before creating a left-sided access, rather than only after symptoms appear, should identify patients at higher risk of early outflow failure. No study has tested this sequence, and current guidance does not require preoperative central venous cross-sectional imaging.

#### 3.2.4 Adverse hemodynamic environment caused by anatomical vulnerability

Direct and near-direct studies establish the LBV’s long retrosternal course, focal sternum–aortic compression, multi-axial angulation, and distinct lateral and medial junctions (Hammer et al., 2000; Salik et al., 2007; Yakushiji et al., 2009; Vertemati et al., 2020). These studies do not measure LBV time-averaged wall shear stress (TAWSS), oscillatory shear index (OSI), relative residence time (RRT), or vortex structure. General vascular and AVF studies provide mechanistic plausibility that geometry and flow perturbation can generate low or oscillatory shear and spatial gradients (Papaioannou et al., 2006; Ene-Iordache and Remuzzi, 2012; Franzoni et al., 2016). The defensible conclusion is that the anatomy creates candidate conditions for adverse flow and external loading, not that a continuous chain from compression to wall shear stress (WSS) to intimal hyperplasia has been demonstrated in the LBV.

Even without overt iatrogenic injury, the LBV may be mechanically vulnerable because of its congenital configuration. Resistance to focal compression in the retrosternal segment should therefore be evaluated explicitly rather than extrapolated from iliofemoral concepts addressing circumferential recoil.

### 3.3 Second hit: Iatrogenic injury and the central role of central venous catheters

Central venous catheters, particularly the tunneled cuffed catheters commonly used for HD, are the clearest iatrogenic factor driving central venous disease. Recognized risk processes include mechanical endothelial injury, local flow disturbance, catheter dwell time, and cumulative exposure from repeated catheterization, and in the LBV they should be interpreted together with the left-sided course and angulated geometry.

Catheter–wall friction and stiffness–compliance mismatch are engineering hypotheses requiring trajectory-specific bench or finite-element testing and lesion-localized validation.

An indwelling catheter can plausibly alter the local velocity field and near-wall shear, and the general endothelial literature supports biological responses to disturbed flow (Chiu and Chien, 2011). However, the cited literature does not directly demonstrate catheter-induced stagnation, vortices, or low-shear zones in the LBV. These features should therefore be treated as candidate outputs of catheter-inclusive computational fluid dynamics (CFD) or flow-loop studies, rather than as an observed step in an LBV pathological sequence.

The catheter route also matters. Left internal jugular catheterization for HD has been associated with higher rates of inadequate blood flow and ipsilateral access complications than right-sided access (Salgado et al., 2004), and three-dimensional reconstructions show the longer, more angulated left central venous route (Salik et al., 2007). A mechanical explanation involving greater wall contact or friction is plausible, but contact pressure and frictional area were not measured and remain unvalidated engineering hypotheses.

Beyond dialysis catheters, pacemaker/defibrillator leads, peripherally inserted central catheters (PICCs), and infusion ports are associated with central venous obstruction (Gonsalves et al., 2003; Yevzlin, 2008). Occult or asymptomatic central venous lesions are documented clinically, and some later become symptomatic (Taal et al., 2004; Renaud et al., 2012). A case report describes rapid symptom emergence after creation of a new left-sided access in the presence of pre-existing external compression (Yamaguchi et al., 2020). These observations support an unmasking hypothesis but do not quantify a flow threshold.

### 3.4 Third hit: abnormal hemodynamic stress

#### 3.4.1 The double-edged effect of arteriovenous access

A mature AVF/AVG is the lifeline for maintenance HD, but access creation produces a large increase in venous flow and alters the shear environment. This is supported by AVF/AVG remodeling and hemodynamic studies rather than by direct measurements in the LBV (Lu et al., 2014; He et al., 2025; Ninno et al., 2025).

The evidence supports three separate statements that should not be collapsed into a single citation bundle. First, arteriovenous access changes flow and shear and is associated with subsequent remodeling in non-LBV access segments (Lu et al., 2014; He et al., 2025; Ninno et al., 2025). Second, the LBV has complex curvature and junction geometry, but whether high access flow produces vortices, high OSI, or prolonged residence there remains an LBV-specific hypothesis (Salik et al., 2007; Vertemati et al., 2020). Third, occult central venous stenoses exist and may later become clinically important, although the supporting clinical studies did not measure these hemodynamic metrics (Taal et al., 2004; Renaud et al., 2012; Yamaguchi et al., 2020). High flow is therefore a potential amplifier or unmasking factor rather than an isolated, sufficient cause.

#### 3.4.2 WSS pattern and endothelial phenotype: unidirectional pulsatile versus oscillatory bidirectional shear

High access flow should not be equated with uniformly low wall shear stress or prolonged residence time. Increased flow may elevate bulk velocity and wall shear in relatively straight regions, whereas LBV curvature, focal compression, venous junctions, stent edges, struts, and local deformation may simultaneously generate spatially confined flow separation, recirculation, low or oscillatory wall shear stress, and delayed washout. High-flow exposure and regional disturbed-flow metrics may coexist within the same anatomy. Their spatial distribution and biological relevance in the stented LBV remain hypotheses requiring anatomy-specific computational, experimental, and longitudinal validation (Ene-Iordache and Remuzzi, 2012; Franzoni et al., 2016).

The mechanobiology literature implicates disturbed shear broadly, not low WSS in isolation. Low or near-stagnant WSS at recirculation and stent-edge zones is one recognized pattern, whereas abnormally high or spatially discontinuous shear at struts, jets, and stenotic-throat regions is another, so the spatial WSS gradient across these transitions is relevant in addition to WSS magnitude (Ene-Iordache and Remuzzi, 2012; Franzoni et al., 2016). Venous-stent and AVF models further indicate that an established stenosis or ISR can remodel the flow field and reduce portions of both low- and high-WSS surface area, making the relationship potentially bidirectional and non-monotonic (Torikai et al., 2026; Zhang et al., 2026). P2 should consequently report TAWSS distribution, OSI, RRT, spatial WSS gradient, recirculation or washout, branch-flow distribution, and pressure loss together; because RRT is derived from TAWSS and OSI, it should not be an independent co-primary endpoint. The goal is movement toward a flow-matched reference envelope, not maximization of WSS or mandatory improvement over the diseased pre-intervention shear field.

General arteriovenous-access pathology and mechanobiology studies suggest that abnormal shear environments may be associated with endothelial dysfunction, inflammatory-mediator release, smooth-muscle-cell proliferation, neointimal hyperplasia, and thrombus organization (Roy-Chaudhury et al., 2006; Franzoni et al., 2016). The third hit therefore remains a mechanism requiring validation: high flow in the distinctive anatomical setting of the LBV may amplify oxidative stress, inflammatory activation, and mechanical remodeling, but demonstrating this chain requires LBV-specific histology, animal models, or prospective imaging–flow-dynamics studies.

Evidence-proximity note. Direct LBV evidence supports the anatomical-compression phenotype, whereas catheter/device associations and high-flow unmasking draw on Adjacent HD-CVS evidence, and adverse-shear and remodeling links remain Analogous engineering evidence or Mechanistic hypotheses.

## 4 Failure phenotypes and current intervention boundaries: defining unmet device needs

### 4.1 Overall landscape of endovascular therapy and indication principles

This section extracts failure-boundary evidence from current therapies. Intervention is generally reserved for symptomatic LBV stenosis/occlusion with both clinical indicators and imaging/angiographic stenosis (Renaud et al., 2012; Echefu et al., 2023; Lok et al., 2020; Tabriz and Arslan, 2022). For the device-design argument, PTA, BMS, and SG are interpreted by the failure phenotypes they reveal: elastic recoil, inadequate or excessive radial support, focal compression-related deformation, ISR and patency decay, edge stenosis, branch coverage, and dependence on repeated reintervention.

PTA provides foundational boundary evidence: it restores lumen acutely but is limited by elastic recoil and early recurrence in central veins, and comparative evidence between PTA and primary stenting remains inconsistent (Echefu et al., 2023; Wu et al., 2020). Current evidence does not justify routine primary stenting as a standard LBV pathway; in patients with marked extrinsic compression, earlier stent support is best framed as an anatomy-guided research scenario, and short-term luminal support should not be assumed to translate into durable patency.

Etiological heterogeneity may nonetheless shape how device performance is interpreted in the LBV. Wan et al. reported similar primary patency after PTA and SG placement and higher secondary patency after SG placement, although prior catheterization history and balloon diameter differed between groups (Wan et al., 2025). Extrinsic-compression subtypes may therefore depend more on mechanical support, but these data do not establish primary stenting as broadly superior to systematic PTA with selective stenting. Results from mixed thoracic central venous cohorts should be read as general boundary evidence rather than LBV-specific proof.

For this Review, symptoms and access function, etiological subtype, extrinsic compression, prior catheterization, occlusion complexity, junction-specific branch risk, and future access resources are boundary conditions for device evaluation rather than treatment indications. LBV device evaluation should therefore rest on the three-dimensional course, the retrosternal compression window, prior catheter or device exposure, junction-specific branch ostia, landing-zone feasibility, access-flow status, and the cost of consuming future access resources, as organized in Figure 4 and Table 3. Stenosis percentage and immediate post-PTA luminal gain alone do not capture these constraints.

**FIGURE 4:**
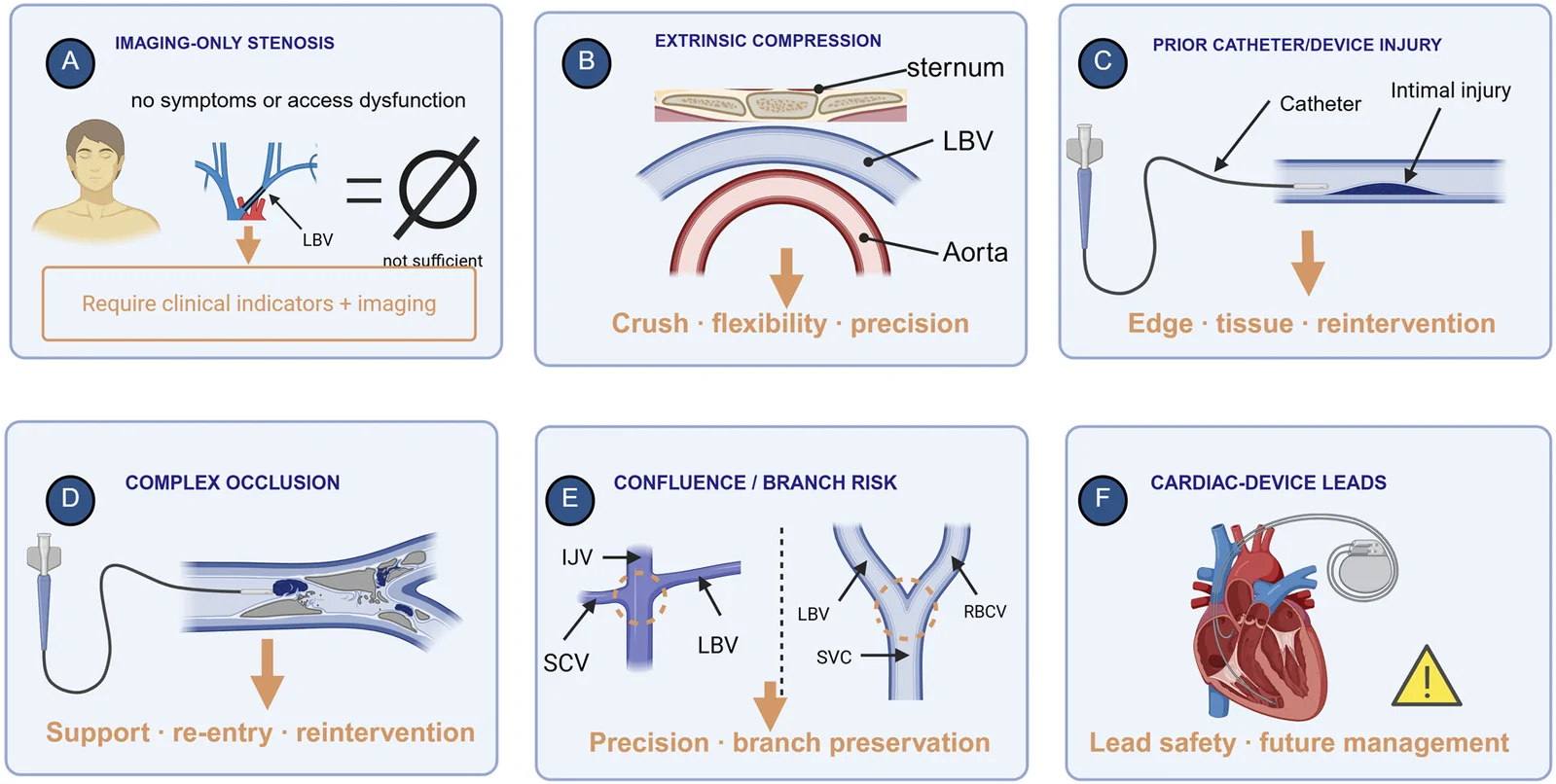
Clinical boundary conditions translated into device-validation constraints for hemodialysis-related left brachiocephalic vein and central venous stenosis. Each panel pairs an independent clinical or anatomical context with the principal device-validation focus it reveals. **(A)** Imaging-detected stenosis without symptoms or access dysfunction defines a clinical-eligibility boundary: imaging severity alone should not be treated as evidence of device efficacy. **(B)** Marked retrosternal sternum–aortic compression without a catheter history motivates evaluation of focal anterior–posterior crush resistance, flexibility, and deployment precision. **(C)** Prior catheter- or device-related venous injury motivates assessment of edge behavior, tissue ingrowth or thrombus organization, and durability under repeated intervention. **(D)** Long-segment or complex occlusion, including failure of conventional guidewire crossing, defines a high-risk boundary for evaluating post-recanalization support, re-entry, and repeat-intervention feasibility rather than a general treatment pathway. **(E)** Lesions near the lateral venous angle or medial brachiocephalic–superior vena cava confluence require precise length control, low foreshortening, and preservation of branch inflow and future venous access. **(F)** Cardiac-device-lead-related disease requires evaluation of lead entrapment, infection risk, and compatibility with future lead and access management. The six panels are independent and non-sequential; the figure is neither a treatment algorithm, a device indication, nor a product-ranking scheme. Abbreviations: IJV, internal jugular vein; LBV, left brachiocephalic vein; RBCV, right brachiocephalic vein; SCV, subclavian vein; SVC, superior vena cava. Created in BioRender. Jin S. (2026) https://BioRender.com/jycnx0y.

### 4.2 From clinical scenarios to device-constraint translation

Table 3 categorizes LBV/CVS scenarios not to recommend a pathway but to define what a candidate device must tolerate or preserve: clinical-indicator context, extrinsic compression, catheter-related injury, occlusion complexity, junction-specific branch risk, and cardiac device leads, the last lying at the procedural periphery of this Review.

### 4.3 Bare-metal stents: radial support with prominent restenosis

BMS overcome elastic recoil by providing sustained radial support, but long-term patency declines and chronic strut-related mechanical stimulation may promote neointimal hyperplasia (Chawla et al., 2025; Haage et al., 1999; Quaretti et al., 2016). Because most comparisons derive from mixed-segment central venous disease rather than isolated LBV cohorts, these data define failure phenotypes rather than LBV-specific efficacy (Figure 5A).

**FIGURE 5:**
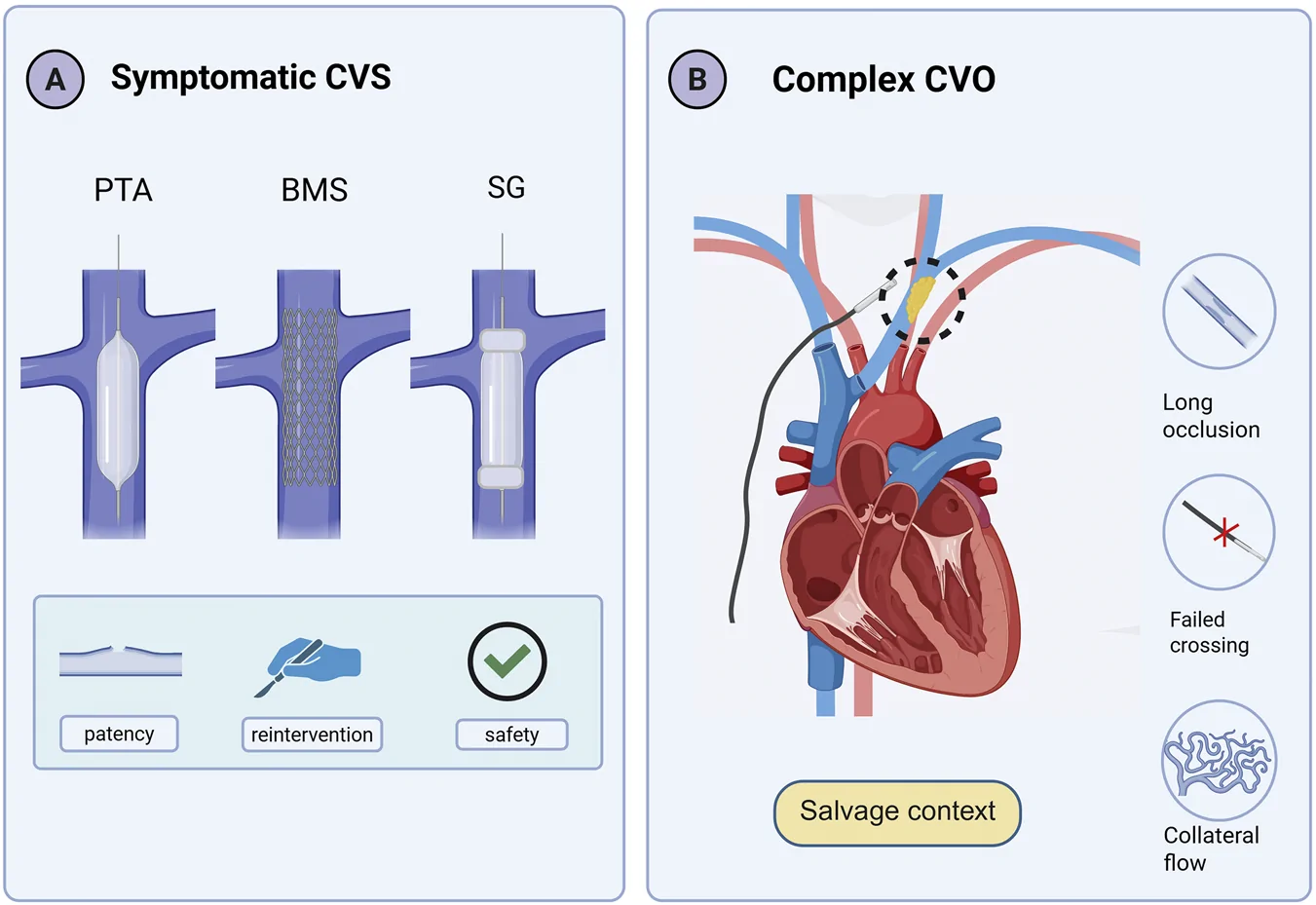
Clinical evidence boundary for intervention in left brachiocephalic and central venous disease. **(A)** Percutaneous transluminal angioplasty (PTA), bare-metal stents (BMS), and stent grafts (SG) are shown as parallel treatment categories reported for symptomatic central venous stenosis. Patency, reintervention, and safety represent shared clinical outcome domains rather than quantitative comparisons or claims of device superiority. **(B)** Long-segment central venous occlusion, failure of conventional guidewire crossing, and extensive collateral flow define a distinct complex central venous occlusion context. Evidence obtained in this salvage setting should not be interpreted as a routine treatment pathway or extrapolated directly to less complex stenosis. Because the available studies vary in anatomy, lesion complexity, intervention technique, outcome definition, and follow-up, the figure defines clinically relevant evidence boundaries but does not rank device classes or establish LBV-specific efficacy. Abbreviations: BMS, bare-metal stent; CVO, central venous occlusion; CVS, central venous stenosis; LBV, left brachiocephalic vein; PTA, percutaneous transluminal angioplasty; SG, stent graft. Created in BioRender. Jin S. (2026) https://BioRender.com/snsg60y.

For long-segment occlusion, lesions not crossed by conventional guidewires, or recurrent thoracic CVO, published experience shifts the analytical focus from single-session reopening to vascular-resource preservation and reintervention planning. Sharp recanalization and complex CVO studies report high technical success, but their adverse-event and reintervention burden defines a high-risk evidence boundary rather than a first-line solution for ordinary LBV stenosis (Figure 5B) (Ashraf et al., 2025; Pasqui et al., 2026).

Comparative studies of BMS versus PTA or SG show limited mid-to long-term advantage for BMS, and ISR remains prominent (Liu et al., 2024; Quaretti et al., 2016). Radial support alone is therefore not a sufficient endpoint for an LBV-oriented stent: it must be evaluated together with crush behavior, axial conformability, branch-safe deployment, and local flow disturbance.

### 4.4 Stent grafts: comparatively stronger evidence but not LBV-specific proof

SG use expanded polytetrafluoroethylene (ePTFE) or similar coverings to block tissue ingrowth through the struts. Their main advantage is improved short-to mid-term patency in selected central venous lesions, although much of this evidence comes from mixed thoracic segments rather than isolated LBV cohorts. Junction-specific risks differ: lateral extension may compromise left internal-jugular or subclavian inflow at the venous angle, whereas medial extension into the SVC may affect right-brachiocephalic inflow or future re-entry. SG therefore raise two design questions: how to avoid transferring proliferative failure to the edge, and how to preserve junctional branches while covering the lesion.

Representative studies show that SG can improve short-to mid-term patency, but 24-month primary patency still declines substantially and multiple stenoses predict worse outcomes (Liu et al., 2024; Quaretti et al., 2016; Jones et al., 2011); LBV-specific data suggest that SG may improve secondary patency compared with PTA (Wan et al., 2025). The covering strategy is promising, but a barrier alone does not resolve long-term restenosis in benign dialysis-related lesions.

Long-term patency in benign lesions, including dialysis-related catheter-associated or extrinsic-compression lesions, is lower than in malignant compressive disease (Chawla et al., 2025). This difference suggests that dialysis-related LBV stent failure may involve more than external compression, with fibrin deposition, thrombus organization, and intimal hyperplasia as additional contributors. Optimization is framed here as a staged pathway that validates mechanical and hemodynamic feasibility before evaluating antithrombotic or antiproliferative concepts.

Key limitations of SG include edge stenosis, intrastent thrombosis or thrombus organization, permanent coverage of important branches, larger delivery-system profiles, and insufficient data on covering fatigue and aging in the LBV environment (Tabriz and Arslan, 2022). Covering strategies should be assessed within a broader biomaterial-device system that includes edge design, branch preservation, fabric fatigue, hemocompatibility, and reintervention feasibility.

### 4.5 Shared stent-failure phenotype: ISR and patency decay

ISR is a core obstacle to mid- and long-term LBV stent durability and the bridge between patency data and design considerations. In maintenance HD patients with central venous occlusive disease, ISR occurred in 36.1% of the 72 patients in whom stenting was technically successful, over a median follow-up of 15 months (interquartile range 10–24 months), with 6-, 12-, and 18-month primary patency declining progressively (Yu et al., 2024). Longer dialysis duration was associated with ISR, and the meta-analytic gap between primary and assisted-primary patency, rated very low certainty, indicates that long-term patency often depends on surveillance and reintervention, constraining expectations rather than establishing effect sizes (Chawla et al., 2025). Luminal restoration is therefore only a starting point.

### 4.6 Major limitations of current evidence

Existing clinical evidence has four major limitations: most studies are retrospective and of low level; larger studies pool brachiocephalic, subclavian, and SVC lesions without adequate LBV-specific subgroups; patency definitions, follow-up protocols, and reintervention triggers are inconsistent; and benign dialysis-related lesion data are scarcer than malignant-compression data (Yu et al., 2024; Chawla et al., 2025; Wan et al., 2025; Aronhime et al., 2024). The next step is to translate the observed failure phenotypes into measurable mechanical, hemodynamic, and biomaterial validation targets.

## 5 From failure phenotypes to validation targets: mechanistic bottlenecks of ISR

Using the evidence-proximity framework defined above, this section converts recurrent failure phenotypes into candidate validation targets. LBV in-stent restenosis (ISR) is interpreted at three descriptive mechanism levels, denoting position in the mechanism-to-validation framework rather than evidence-quality grades: observed clinical and histological phenomena; hemodynamic and stent-mechanics extrapolations; and downstream biological hypotheses. The aim is to link patency failure to measurable engineering domains—compression, deployment precision, branch preservation, fatigue, and flow disturbance—while keeping molecular signaling as a future mechanism-generating layer.

### 5.1 Level 1: phenomena observed in dialysis/CVS/LBV and venous stent pathology

Direct clinical evidence supports LBV features such as a long transverse course, multi-axial angulation, retrosternal pre-aortic-arch compression, PTA-related elastic recoil, stent compression deformation, ISR, and patency decay (Vertemati et al., 2020; Itkin et al., 2004; Wan et al., 2024; Yu et al., 2024; Chawla et al., 2025; Hammer et al., 2000; Wan et al., 2025). These data support the statement that the LBV is a distinctive central venous segment with high reintervention risk, and they provide the clinical foundation for candidate design validation.

### 5.2 Level 2: mechanisms extrapolated from venous stents, AVF models, and computational studies

Indirect mechanistic evidence suggests that low WSS, high OSI/RRT, microvortices downstream of stent struts, and low-velocity recirculation zones at stent edges may be candidate explanations for the spatial localization of neointimal hyperplasia (Williams et al., 2023; Liao et al., 2024; Franzoni et al., 2016; Ene-Iordache and Remuzzi, 2012). AVF/AVG, computational fluid dynamics, and venous stent studies support associations among complex geometry, focal low shear, pro-inflammatory adhesion-molecule expression, thrombus organization, intimal thickening, and vascular-wall remodeling, but these remain extrapolations for the post-stent LBV setting.

Stent-to-vein compliance mismatch is a separate candidate solid-mechanics pathway at the landing zones. A relatively stiff stented segment adjacent to compliant native vein may concentrate deformation and wall stress at the transition; this differs from the WSS/OSI/RRT pathway and is supported mainly by arterial rather than LBV evidence (Berry et al., 2002). In a thin-walled, low-pressure vein, the relative stiffness contrast may be proportionally larger than in the arterial settings from which this concept derives, although the absolute wall-stress change and its clinical contribution remain to be measured. The corresponding P1 endpoints are the axial compliance or incremental-stiffness profile and its gradient across each stent edge. This pathway remains within the D1 solid-mechanics domain, alongside H1, rather than forming a separate hypothesis.

The biological responses summarized in Figure 1 should not be read as a universal sequence in which endothelial injury necessarily causes thrombosis, followed by thrombus organization and neointimal hyperplasia. A thrombotic-organizing route and a proliferative-remodeling route may occur independently, in parallel, sequentially, or with reciprocal interaction; either may contribute to lumen re-narrowing, ISR, and patency loss, and their relative contributions may vary with anatomy, implantation conditions, lesion history, and follow-up stage. This interpretation yields design questions such as low-disturbance mesh architecture, edge-shape optimization, and patient-specific computational fluid dynamics and fluid–structure interaction (FSI) assessment, but does not show that any configuration will reduce ISR in LBV patients.

Mechanistically, thrombus organization begins with a platelet-fibrin thrombus, typically precipitated by stasis, catheter contact, or acute flow disruption, which is progressively infiltrated by fibroblasts and recanalized; it is a repair response to a thrombotic event. Neointimal hyperplasia is a smooth-muscle-cell- and matrix-driven response to wall injury or sustained abnormal shear and can develop without an antecedent thrombus. The two can co-occur and interact, but a stented segment can develop one without the other, and their histological and imaging signatures differ.

### 5.3 Level 3: downstream biological hypotheses for future validation

Detailed molecular candidates are summarized in Supplementary Box S1, and their evidence proximity is mapped to specific claims in Supplementary Table S6. Candidate couplings include mechanosensitive signaling through Piezo1 and inflammasome activation (Shinge et al., 2021; Shinge et al., 2022; Zeng et al., 2025), together with a reactive-oxygen-species/NF-κB axis through which disturbed flow and cyclic external compression could amplify inflammatory signaling (Chandel et al., 2000; Reynaert et al., 2006; Helmcke et al., 2009). None has been demonstrated in LBV in-stent tissue; they are therefore treated only as downstream hypotheses that may connect mechanical loading with inflammation, migration, matrix remodeling, and proliferation. The practical validation focus remains on imaging-visible deformation, fatigue, branch flow, WSS/OSI/RRT patterns, ISR localization, and access-preservation outcomes.

Evidence-proximity note. The principal P1/P2 validation targets are anchored by observed phenomena and Analogous engineering evidence, whereas downstream biology remains a Mechanistic hypothesis to pursue alongside, not before, measurable mechanical and hemodynamic endpoints. Figure 6 integrates observed failure phenotypes, candidate mechanical and hemodynamic mechanisms, and downstream biological hypotheses into interacting routes to ISR. Figure 7 presents the corresponding staged validation roadmap.

**FIGURE 6:**
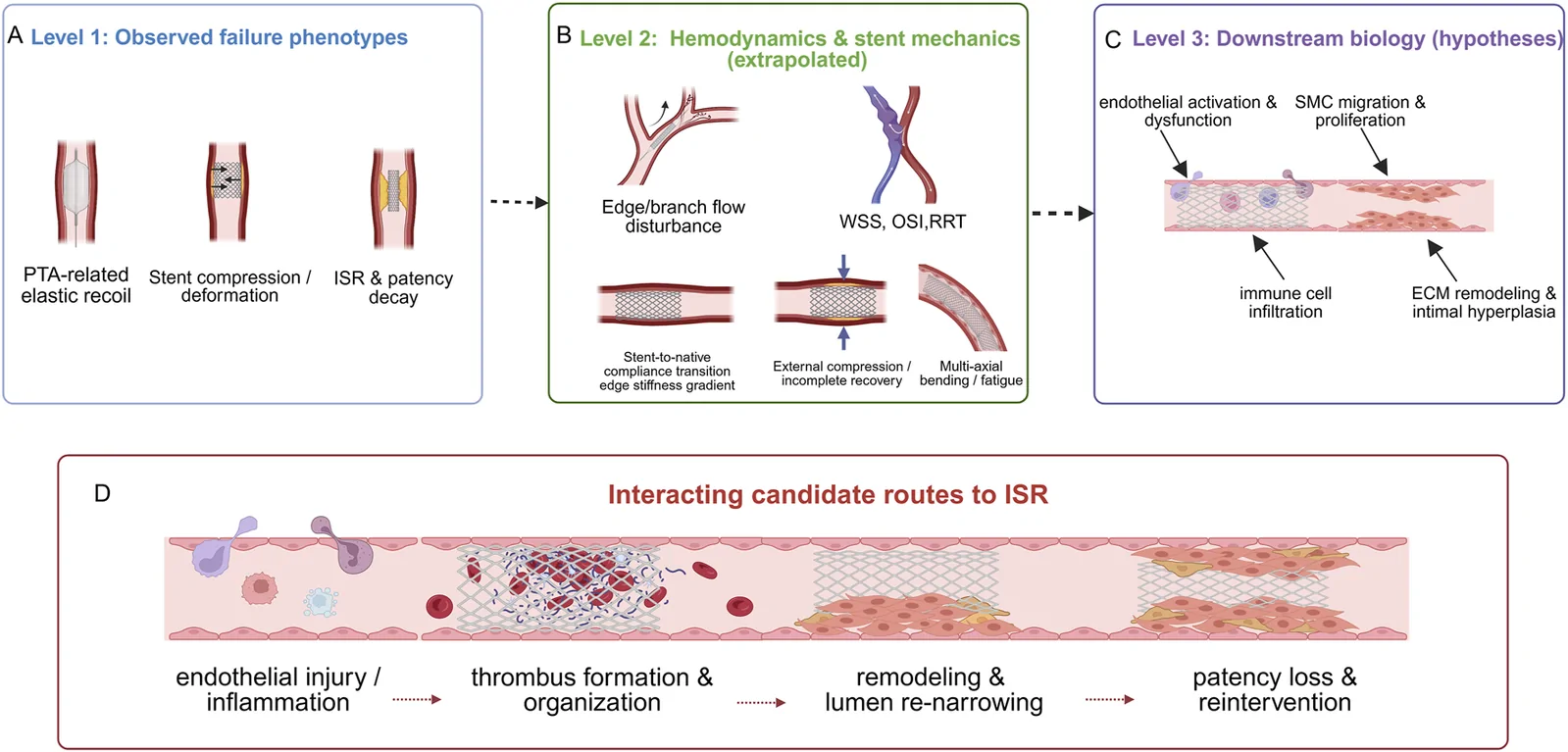
Evidence-layered mechanistic framework for in-stent restenosis in the stented left brachiocephalic vein. **(A)** Level 1 shows observed failure phenotypes: PTA-related elastic recoil, stent compression or deformation, and ISR or patency decay. **(B)** Level 2 presents extrapolated candidate mechanisms: edge or branch flow disturbance; the local hemodynamic environment, characterized by WSS, OSI, and RRT; the stent-to-native-vein compliance transition and edge stiffness gradient; external compression with incomplete recovery; and multi-axial bending or fatigue. The compliance transition is a candidate solid-mechanics pathway distinct from local hemodynamics. Support derives mainly from analogous arterial evidence; this pathway has not been validated for LBV edge hyperplasia. **(C)** Level 3 shows downstream biological hypotheses: endothelial activation or dysfunction, SMC migration or proliferation, immune-cell infiltration, and ECM remodeling or intimal hyperplasia. **(D)** Candidate routes converge on endothelial injury or inflammation, thrombus formation or organization, remodeling with lumen re-narrowing, and patency loss or reintervention. They may occur independently, in parallel, or interactively; thrombus formation is not assumed to precede intimal hyperplasia. All panels are schematic and do not depict computed flow fields. Levels indicate evidence proximity and conceptual role rather than causal certainty or temporal order; arrows denote proposed relationships. Abbreviations: ECM, extracellular matrix; ISR, in-stent restenosis; LBV, left brachiocephalic vein; OSI, oscillatory shear index; PTA, percutaneous transluminal angioplasty; RRT, relative residence time; SMC, smooth muscle cell; WSS, wall shear stress. Created in BioRender. Jin S. (2026) https://BioRender.com/xz8i78v.

**FIGURE 7:**
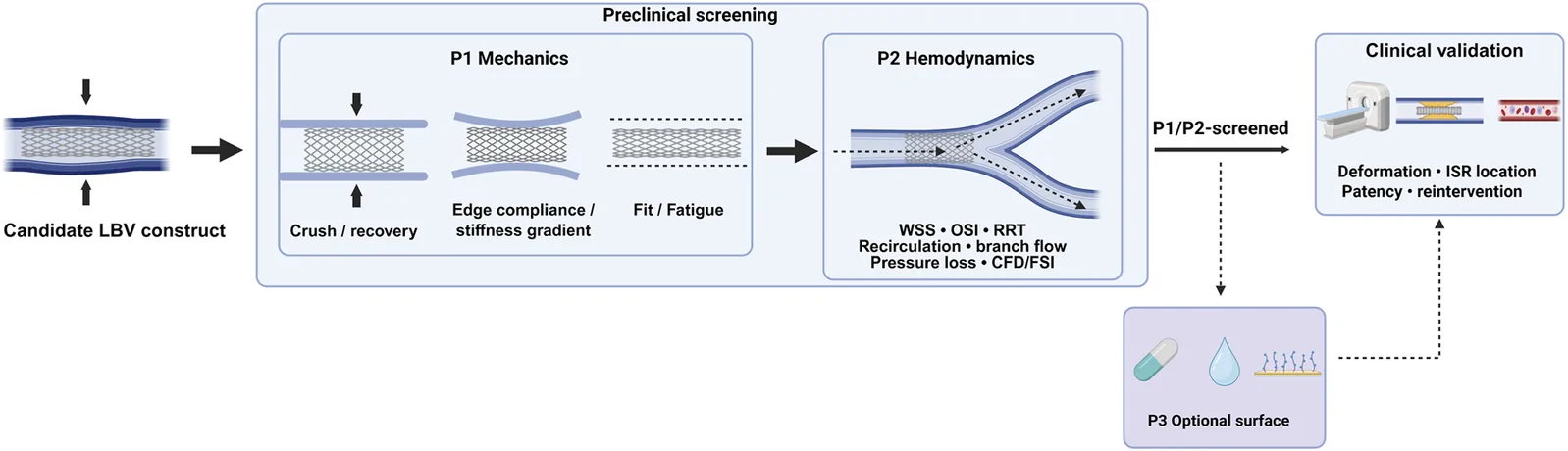
Stage-gated validation roadmap for an anatomy-informed left brachiocephalic vein stent platform. A candidate left brachiocephalic vein (LBV) construct first undergoes preclinical screening in P1, which evaluates focal crush and post-unloading recovery, the edge compliance or incremental-stiffness gradient at both stent-to-native-vein transitions, and anatomical fit and fatigue; for covered constructs, cover–frame integrity and covering fatigue are required P1 components. Constructs meeting P1 criteria proceed to P2, which evaluates WSS, OSI, RRT, recirculation, branch flow, and pressure loss using CFD, FSI, and, where feasible, experimental flow testing. “P1/P2-screened” denotes completion of the proposed screening stages and does not imply clinical validation, efficacy, or superiority. P3 is an optional drug, active coating, or functional surface shown as a dashed branch; any modification that alters mechanics or flow requires repeat evaluation of the affected domain. Baseline thrombogenicity, hemocompatibility, tissue compatibility, and structural integrity remain mandatory. Clinical validation relates post-implant deformation, ISR localization, patency, and reintervention to the screening stages and is a later correlation step, not a P2 release criterion. Arrows denote a validation sequence, not a clinical treatment algorithm. Abbreviations: CFD, computational fluid dynamics; FSI, fluid–structure interaction; ISR, in-stent restenosis; LBV, left brachiocephalic vein; OSI, oscillatory shear index; P1–P3, validation stages 1–3; RRT, relative residence time; WSS, wall shear stress. Created in BioRender. Jin S. (2026) https://BioRender.com/ow9q2hz.

## 6 Biomaterial and venous-device constraints: P1/P2/P3 evaluation requirements for LBV-oriented stent concepts

To make the framework testable rather than descriptive, we state two falsifiable hypotheses that organize the validation agenda. H1 (primary, mechanical): in the LBV, resistance to focal unidirectional crush within the sternum–aortic compression window may be a more important determinant of stent deformation, durability, and the spatial localization of in-stent restenosis than circumferential radial resistive force alone. H1 is comparative rather than absolute: it does not claim that radial resistive force is irrelevant, but that focal crush metrics carry information about LBV performance that radial metrics do not. It is approachable through LBV-specific unidirectional-compression and multi-axial bending-fatigue bench testing, finite-element analysis reproducing patient-derived curvature and end constraints, and spatially resolved imaging–patency correlation. It would be weakened, and at the limit rejected, if circumferential radial resistive force predicted deformation, loss of lumen, and ISR location at least as well as focal crush metrics once material, diameter, oversizing, and deployment geometry were matched.

H2 (secondary, hemodynamic): after controlling for achieved lumen, access-flow state, anatomical phenotype, and deployment geometry, stent architecture and edge configuration generate spatially reproducible regions of disturbed flow, including low or oscillatory shear, steep spatial wall shear stress (WSS) gradients, persistent edge recirculation, and prolonged residence. Early post-implant hemodynamic abnormalities are hypothesized to add incremental prediction of the later spatial distribution of ISR beyond anatomical and procedural covariates. H2 therefore requires post-implant geometry and flow acquired before appreciable ISR and spatially registered with later ISR imaging. The pre-intervention stenotic flow field provides mechanistic context but is not the P2 acceptance comparator. H2 would be rejected if early hemodynamic metrics failed to predict later ISR beyond residual stenosis, access flow, anatomy, and deployment characteristics.

A design prediction from H1 is that, with material, diameter, length, oversizing, and test conditions matched, a functionally zoned concept that decouples its end-anchoring zones from a central crush-resistant segment will improve the residual-diameter-versus-flexibility trade-off relative to a uniform-topology prototype. This prediction requires endpoint-wise testing and cannot be inferred from a single radial-force or crush value.

The supporting evidence for each hypothesis is indirect and motivating rather than confirming. For H1, the most relevant bench dataset compared seven venous stents and found that crush resistance and radial resistive force do not order the devices identically. The Wallstent showed the highest crush resistance between 90% and 60% of nominal diameter, whereas the Sinus-Obliquus had the highest radial resistive force and also the highest crush resistance at 50% of nominal diameter (Dabir et al., 2018). Two qualifications matter. The divergence is compression-level dependent rather than uniform, so the two are related but non-redundant descriptors, not opposites. Because they were obtained on different apparatus under different loading geometries, their absolute magnitudes should not be compared as a ratio, so only within-study ordering is informative.

For H2, AVF/AVG and bifurcation/curvature studies associate disturbed shear with stenosis-prone regions, but recent venous-stent and AVF modeling shows why timing and comparator choice matter: an ISR-containing geometry can obscure the earlier shear pattern, and lesion growth can shift both high- and low-WSS regions toward different ranges (Ene-Iordache and Remuzzi, 2012; Franzoni et al., 2016; Torikai et al., 2026; Zhang et al., 2026). No LBV-specific longitudinal study has tested this directly, so both hypotheses remain open.

These hypotheses map onto the triple-hit model introduced earlier (Figures 1, 2), which describes the same failure process at a different level of formalization. The first hit, anatomical vulnerability and extrinsic compression, is the mechanical exposure that H1 formalizes as focal unidirectional crush loading; the second and third hits, catheter-related injury and high-flow hemodynamic stress, converge on the disturbed-shear and residence-time environment that H2 formalizes and P2 evaluates. The Level-3 downstream biological hypotheses (Supplementary Box S1) sit downstream of both and may motivate an optional P3 layer without independently driving the design framework.

The organizing question in what follows is not which available device is best, but which measurable constraints a future LBV-oriented stent concept must satisfy. Anatomy-specific boundary conditions define failure phenotypes, failure phenotypes define material, structural, deployment, and flow constraints, and these constraints are assigned to P1, P2, and the optional P3. Each constraint is stated with the material family it applies to, the mechanical or flow mode that produces it, and the endpoint that would measure it.

### 6.1 Biomaterial families and device-performance logic

For an LBV-oriented venous stent, the biomaterial question is not only whether the device reopens a stenotic lumen, but whether the scaffold, covering, or surface tolerates the LBV compression-and-junction environment without creating new failure modes. Three device-material families can be distinguished for validation purposes. First, uncovered nitinol or metallic scaffolds are primarily mechanical-conformability systems. Their key endpoints include radial resistive force (RRF), chronic outward force (COF), unidirectional crush resistance, bending and torsion fatigue, foreshortening, deployment precision, and re-entry feasibility (Dabir et al., 2018; Lim and Black, 2022; Schwein et al., 2018; Maleckis et al., 2018; Maleckis et al., 2017; Cheng et al., 2020). Within this family, manufacturing method is itself a design variable. Laser-cut scaffolds tend toward uniform strut geometry and higher circumferential stiffness, whereas braided or woven scaffolds tend toward greater axial flexibility, with foreshortening and crossing-point wear as distinct failure modes; venous-specific comparisons report different luminal-change patterns between them (Liao et al., 2024). Since neither has been tested under LBV-specific unidirectional compression, future reporting should identify both alloy and manufacturing method rather than treat “self-expanding metallic stent” as one category.

Second, covered stent grafts add a polymeric barrier such as ePTFE. Their key endpoints include barrier integrity, membrane fatigue, edge stenosis, branch compromise, hemocompatibility, thrombosis, and preservation of future access resources (Lok et al., 2020; Liu et al., 2024; Anaya-Ayala et al., 2011; Ginsburg et al., 2015; Eadie et al., 2014; Lin et al., 2016).

Third, functionalized, drug-delivery, or surface-modified concepts add biological performance claims. Their endpoints must include release behavior under venous flow, coating integrity after crimping and deployment, platelet and coagulation activation, endothelial and smooth-muscle-cell response, and long-term safety (Williams et al., 2023; Eadie et al., 2014; Lin et al., 2016).

Representative published bench-mechanics evidence for these device-material families, and the LBV-specific gap it leaves, is summarized in Table 4.

**TABLE 4:** Published venous-stent bench-mechanics evidence and the LBV-specific validation gap. All values are taken from published *in vitro* studies and are reorganized for comparison; no pooled analysis, cross-study ranking, or P1 acceptance benchmark is implied. Percentages in parentheses denote the residual (nominal) stent diameter at which the value was recorded, not the percentage of diameter reduction. Radial resistive force, chronic outward force, and crush resistance were measured on different apparatus under different loading geometries; only within-study orderings are interpretable, and absolute magnitudes should not be compared across quantities. The devices differ in material, construction, and labeled indication; product- and market-specific regulatory status is summarized in Supplementary Table S1. Abbreviations: COF, chronic outward force; FEM, finite-element method; ISR, in-stent restenosis; RRF, radial resistive force; WSS, wall shear stress.

Mechanical quantity/source	Representative published value	Implication for the LBV	Evidence boundary
Radial resistive force (RRF) (Dabir et al., 2018)	Highest of seven venous stents tested: Sinus-Obliquus, 19.41 N/cm	Reflects resistance to uniform circumferential compression; high RRF does not imply resistance to focal crush	Iliocaval bench data; not directly transferable to the LBV
Chronic outward force (COF) (Dabir et al., 2018)	Highest COF at 50% and 30% residual nominal diameter: Sinus-Obliquus, 7.93 and 16.97 N/cm; highest COF at 90% and 80% residual nominal diameter: Venovo, 4.83 and 5.37 N/cm	Excessive COF may chronically stimulate the venous wall and promote intimal hyperplasia	Iliocaval bench data; not LBV-specific
Crush resistance (Dabir et al., 2018)	Highest crush resistance from 90% to 60% residual nominal diameter: Wallstent, 0.46–1.16 N/cm; highest crush resistance at 50% residual nominal diameter: Sinus-Obliquus, 1.41 N/cm	Crush resistance and RRF do not order the same stents identically, and the divergence is compression-level dependent; this is consistent with, but not sufficient to establish, H1 (focal crush carrying information that RRF alone does not)	Parallel-plate compression on a separate test rig; not LBV unidirectional sternum–aortic loading, and not numerically comparable with RRF/COF values
End-fixation effect (Dabir et al., 2018)	Wallstent RRF and COF depend strongly on whether the stent ends are constrained	Supports mechanically decoupling end-anchoring zones from a central crush-resistant segment (H1 corollary)	Bench simulation; requires LBV anatomical validation
Multi-mode deformation (Maleckis et al., 2017; Maleckis et al., 2018)	Femoropopliteal nitinol stents behave differently under compression, tension, bending, and torsion	LBV testing should report compression, bending, torsion, and fatigue separately, not a single radial value	Femoropopliteal artery; extrapolated evidence
FEM mechanical assessment (Yu et al., 2024)	FEM used to relate stent mechanics to ISR in central venous occlusive disease	Provides a methodological precedent for the H1 test path in an adjacent setting	Not LBV-specific; needs patient-specific geometry
Mechanical-characteristics reviews (Lim and Black, 2022; Schwein et al., 2018)	Venous stents must jointly balance radial force, crush resistance, and conformability	Frames P1 as joint evaluation of support, crush resistance, and conformability	Qualitative reviews

No published dataset has tested a stent under LBV-specific unidirectional (sternum–aortic) compression with patient-derived curvature and a junction-adjacent short landing zone. Iliofemoral, femoropopliteal, and SVC evidence can identify candidate devices and test methods but cannot define an LBV “best” benchmark, so P1 should use a prespecified comparator panel selected for indication relevance and complementary mechanical profiles.

A methodological distinction governs how the commercial device data in Table 4 and Supplementary Table S1 should be read. The RRF, COF, and crush-resistance comparisons across Wallstent, Sinus-Obliquus, and Venovo come from a single study applying a consistent bench protocol (Dabir et al., 2018), which supports within-study comparison. By contrast, the clinical patency figures cited for different devices (e.g., Zilver Vena in Comerota et al., 2024; Abre in Aronhime et al., 2024; Venovo thoracic experience in Maleux et al., 2024) come from separate studies with different populations, lesion mixes, catheter histories, and follow-up protocols. They are not head-to-head comparable, and each is cited only for the design implication it illustrates.

This family-based framing prevents overgeneralization. A stent graft may improve tissue-ingrowth control yet fail through edge stenosis, branch coverage, or covering fatigue; a highly flexible uncovered scaffold may conform well to curvature yet fail to resist focal osteoarterial compression; and a functionalized concept may be biologically attractive yet cannot compensate for poor deployment precision, branch compromise, or persistent flow disturbance. Biomaterial innovation should therefore be evaluated as part of an integrated device–environment system.

### 6.2 Compression resistance and radial force control: countering extrinsic compression without excessive stimulation

Translating mechanisms into device considerations first requires distinguishing radial resistive force (RRF), chronic outward force (COF), and crush resistance, because the core LBV scenario combines sternum–aortic arch compression, multi-axial angulation, and end-anchoring coupling, and cannot be judged by radial support alone (Dabir et al., 2018; Lim and Black, 2022; Schwein et al., 2018).

These three quantities are not mechanically interchangeable, because they are measured under different loading geometries and boundary conditions. RRF is measured by uniform radial compression around the entire circumference and characterizes resistance to symmetric narrowing. COF is the outward force exerted against a boundary when the stent is held below nominal diameter, and characterizes chronic wall loading rather than resistance to an applied load. Crush resistance is measured under localized or line loading over part of the circumference and characterizes asymmetric, focal deformation, in which failure more often involves local strut buckling and ovalization than uniform collapse. Recovery after unloading is a separate property again, since a stent may resist a given crush load yet fail to regain its lumen (Figure 3C). Because the loading geometries differ, RRF ordering need not predict crush ordering, which is the mechanical basis for the partially divergent rankings in Table 4 (Dabir et al., 2018) and the rationale for H1: the sternum–aortic window applies focal, largely unidirectional loading over a limited arc of the circumference, the geometry that crush resistance characterizes and RRF does not. It is also why residual diameter, crush load, and post-unloading recovery should be reported separately rather than summarized as a single stiffness value.

A simple analytical contrast makes this explicit. For a thin circular ring of flexural rigidity *EI* and radius *R*, uniform external pressure produces collapse as an elastic bifurcation, at the classical mode-2 critical pressure $p_{cr}=\frac{3EI}{R^{3}}$ per unit length, so the ring is stable up to *p*_cr_ and then loses stiffness abruptly (Timoshenko and Gere, 1961). Under two diametrically opposed radial line loads *F*, the same ring instead responds by continuous local bending; the bending moment scales with FR and depends on the loaded arc (Young and Budynas, 2002). Two consequences follow. First, the two loadings produce different limiting mechanisms: an elastic buckling instability in one case, progressive local bending, strut buckling, or fracture over a contact arc in the other. Second, the focal response is governed by the moment distribution over the loaded arc, so the width of that arc, a property of the sternum–aortic window rather than the device, enters the problem explicitly, whereas it does not appear in *p*_cr_ at all. Ranking devices by a circumferential metric therefore carries no guarantee about their ranking under focal loading unless both are governed by the same limiting mechanism. A deployed stent is a helical or cellular structure, not a homogeneous ring, so these expressions only make the difference between loading geometries explicit; they do not predict absolute device performance.

For superelastic nitinol scaffolds, the transformation plateau, hysteresis, and loading history alter effective stiffness and unloading recovery; the tangent stiffness on the transformation plateau is substantially lower than the linear-elastic modulus assumed by the ring idealization. Focal compression may therefore progress through local strut bending or ovalization rather than the abrupt bifurcation predicted for a linear-elastic ring. This nonlinearity reinforces the need to test circumferential and focal loading separately (Maleckis et al., 2018).

Bench comparisons and venous-stent engineering reviews suggest that devices optimized for the iliofemoral environment may not translate directly to the LBV (Dabir et al., 2018; Smith et al., 2025; Lim and Black, 2022; Schwein et al., 2018); brand-level bench values are given in Supplementary Table S1. The main design implication, itself requiring LBV-specific validation, is that future platforms may need to mechanically decouple the end-anchoring zones from the central functional segment while accounting for stent–vessel-wall boundary coupling.

This premise should be situated relative to its closest existing paradigm. Zilver Vena, Venovo, and Sinus-Obliquus were developed or evaluated predominantly for iliac vein compression (May–Thurner-type) and post-thrombotic iliofemoral outflow obstruction, settings in which non-uniform, compression-dominant loading is already a recognized design problem (Comerota et al., 2024; Dabir et al., 2018). Regulatory status varies by jurisdiction. The statements below refer specifically to U.S. labeling. In the United States, specified Wallstent configurations are indicated for improving central venous luminal diameter after unsuccessful angioplasty in chronic-hemodialysis patients with innominate or subclavian venous-outflow stenosis. Venovo, Abre, and Zilver Vena are indicated for symptomatic iliofemoral venous-outflow obstruction, whereas Sinus-Obliquus is included here only as a bench comparator. For devices whose labeled anatomy is limited to the iliofemoral veins, LBV use is off-label, and the cited iliofemoral bench and clinical findings are anatomical extrapolations rather than LBV-specific evidence; off-label use of an approved device is distinct from investigational-device status. Product- and market-specific details are provided in Supplementary Table S1 (Boston Scientific, 2026; U.S. Food and Drug Administration, 2019; U.S. Food and Drug Administration, 2020a; U.S. Food and Drug Administration, 2020b). The crush-versus-RRF distinction anchoring H1 is therefore an established premise in iliocaval venous stent engineering rather than a new principle. The contribution here is its site-specific transfer to the sternum–aortic compression window, multi-axial angulation, and junction-adjacent anatomy of the LBV, which differ from the iliofemoral segment in loading geometry, branch constraints, and access-related high-flow exposure.

Radial force is not simply a matter of “higher is better”: excessive support may chronically stimulate the venous wall and promote intimal hyperplasia, whereas insufficient crush resistance cannot withstand osteoarterial compression. Because stent materials and geometries respond differently to circumferential compression, focal crush, axial shortening, bending, torsion, and repeated compression-recovery cycles, LBV testing should report these modes separately rather than substitute a single radial-force metric (Lim and Black, 2022; Schwein et al., 2018; Maleckis et al., 2018; Maleckis et al., 2017). A P1 battery should report residual diameter, load–displacement behavior, shape recovery after unloading, bending stiffness and fatigue, foreshortening, deployment error, and branch-access performance, with each candidate and comparator tested in the final sterilized configuration at matched diameter, length, oversizing, temperature, deployment state, fixture, curvature, and end constraint. Safety-critical outcomes, such as fracture, loss of structural integrity, deployment failure, and irreversible branch obstruction, require absolute acceptance criteria; continuous outcomes require prespecified margins justified from measurement repeatability and clinical relevance, with independent test units, a variance-based sample-size rationale, and confidence intervals. Because no device dominates every property, the full trade-off profile should be reported, for example, as a Pareto comparison across focal-crush resistance, RRF/COF, flexibility, foreshortening, and branch preservation; strength in one domain cannot offset a safety-critical failure.

Recognized standards can anchor test conduct without supplying LBV acceptance thresholds. ISO 25539–2 provides a general vascular-stent framework but does not comprehensively specify venous-stent testing (International Organization for Standardization, 2020). ASTM F3067-26 addresses circumferentially uniform radial loading, so for a self-expanding nitinol LBV concept it informs COF rather than focal crush and is not a parallel-plate or sternum–aortic crush method (ASTM International, 2026). ASTM F2942-25 guides non-radial axial, bending, torsional, and compression durability but leaves mode, amplitude, and cycle count to be justified (ASTM International, 2025). ASTM F2477-24 covers *in vitro* pulsatile durability, that is cyclic diametric distension, and does not substitute for focal external compression (ASTM International, 2024). ASTM F3505-21 specifies kink resistance under single-plane bending only, without simultaneous torsion or external compression (ASTM International, 2021b). ASTM F2081-06(2022) guides dimensional attributes rather than strength (ASTM International, 2022), with complementary guidance on three-point bending (ASTM International, 2021a), endovascular-prosthesis evaluation (International Organization for Standardization, 2017), and graft materials (International Organization for Standardization, 2016). None reproduces the combined sternum–aortic focal load, LBV curvature, respiratory excursion, upper-limb motion, and junction-specific branch constraints, so custom LBV test methods remain necessary.

The scope of these standards can also be stated quantitatively. Over a nominal 10-year implant life, a stent in this segment is exposed to at least four superimposed cyclic regimes differing in frequency, amplitude, and loading geometry. Cardiac-frequency diametric distension accumulates approximately 4 × 10^8^ cycles at a resting rate of 70–75 min^−1^, the regime addressed by pulsatile durability testing. Respiratory modulation of the sternum–aortic interval and of LBV curvature accumulates roughly 6 × 10^6^ to 1 × 10^7^ cycles per year at 12–20 breaths min^−1^, of order 10^8^ over 10 years. No LBV-specific activity rate is available for shoulder-girdle or upper-limb loading. As an illustrative calculation, 10^3^ mechanically relevant movements per day over 10 years would yield 3.65 × 10^6^ activity events. This event count is not a measured number of local stent-strain cycles, because one movement may produce no appreciable local strain or more than one strain cycle. Dialysis-session transitions contribute approximately 1.5 × 10^3^ events over 10 years at three sessions per week, each imposing an acute change in access flow and central venous pressure. The first two regimes differ by only four-to sixfold in cycle count, yet potentially by an order of magnitude in local strain amplitude, because respiratory motion acts on curvature and the compression window rather than diameter alone. A construct qualified solely under cardiac-frequency diametric cycling therefore carries no demonstrated margin against the remaining regimes. The cardiac and respiratory counts are arithmetic projections from published physiological rates, not measurements, whereas the upper-limb count is an explicitly stated illustrative scenario; and the governing amplitudes must be established by LBV-specific dynamic imaging and activity-derived load spectra.

For nitinol constructs, cycle count alone is insufficient for fatigue qualification. Nonlinear finite-element analysis of the deployed configuration should quantify the mean and alternating components of maximum principal strain in the stent at the sternum–aortic contact and other critical locations. These values should be interpreted against a strain-based constant-life diagram (CLD) matched to the alloy, processing condition, surface state, and test temperature. A conventional linear Goodman relation or numerical thresholds from other devices should not be transferred directly (Pelton et al., 2008; Cao et al., 2020).

### 6.3 Flexibility, deployment precision, and junction protection

Multi-axial LBV angulation makes rigid stents prone to straightening, end traction, and local stress concentration, whereas excessive flexibility may fail to counter extrinsic compression. A dedicated LBV design concept would therefore need testing for crush resistance at the compressed segment, axial flexibility in curved segments, and low foreshortening with high deployment precision at the confluence. The general nitinol-stent deformation literature supports evaluating compression, tension, bending, and torsion as distinct mechanical modes under physiologically relevant deformation patterns (Maleckis et al., 2018; Maleckis et al., 2017; Cheng et al., 2020). Specific configurations and short-term comparative data are provided in Supplementary Table S1 (Sulakvelidze et al., 2023; Comerota et al., 2024).

Experience with thoracic central venous stents may inform LBV deployment strategy, anchoring-zone design, and oversizing ratio, but outcomes dominated by malignant lesions or non-LBV segments cannot be extrapolated directly to benign dialysis-related LBV disease (Maleux et al., 2024). Device brand, diameter, length, and patency details are given in Supplementary Table S1. This is Analogous engineering evidence: it can suggest test methods and design variables, but not LBV device performance.

The LBV presents two distinct junction-specific branch-protection challenges. At the lateral venous angle, stent extension may cover the left internal-jugular or subclavian inflow; at the medial brachiocephalic–SVC confluence, extension into the SVC may affect right-brachiocephalic inflow, contralateral access, or re-entry. Landing-zone planning, fenestration or branch preservation, re-entry feasibility, and long-term branch patency are therefore validation constraints rather than procedural instructions, and main-trunk patency alone is not sufficient to establish LBV suitability (Lok et al., 2020; Ginsburg et al., 2015; Theodoridis et al., 2024; Hu et al., 2024).

### 6.4 New-generation dedicated venous stents: early experience

Dedicated venous stents have begun to accumulate clinical data, but their relevance to the LBV should be read through a biomaterial-performance lens. Flexible open-cell, hybrid-cell, and large-bore nitinol concepts may each address different portions of the problem: conformability in curved segments, focal support in compression zones, or improved reintervention options. A small HD CVO series reported high technical success without observed stent fracture, which is compatible with, but does not demonstrate, adequate fatigue performance in this segment (Aronhime et al., 2024; Supplementary Table S1). A retrospective comparison of bare venous and conventional bare stents provides additional CVO outcome data, but sample size, follow-up duration, and LBV-specific data remain limited (Akkakrisee and Hongsakul, 2022). Such experience is therefore design inspiration rather than proof of efficacy. Future studies should not treat “dedicated venous stent” as a single category, but should report structural topology, scaffold material, radial and crush behavior, fatigue profile, deployment accuracy, covering or coating status, and branch-preservation strategy within one validation system.

### 6.5 Anatomical segment differences and stent-type selection

Current guidelines do not provide unified recommendations for stent-type selection across LBV segments. Consistent with the three-segment framework introduced earlier, segment-based clinical practice suggests that mechanical requirements differ along the vein: the lateral segment emphasizes adaptation to dynamic compression, whereas the central segment and SVC confluence emphasize deployment precision, crush resistance, and branch preservation (Tabriz and Arslan, 2022). This supports evaluating nonuniform-topology or functionally zoned designs as candidate concepts rather than established solutions.

### 6.6 Covering integrity and hemocompatibility as base-platform attributes, and optional surface functionalization

Covering and blood-contact properties are a recurring source of confusion in staged device frameworks: they are not optional refinements. For any construct intended for implantation, baseline thrombogenicity, hemocompatibility, and tissue compatibility are mandatory cross-cutting safety requirements, while structural integrity is required within the platform evaluation. For a covered construct, the cover–frame interface, membrane fatigue, and deformation-induced fabric damage are required P1 components, and these safety assessments are distinct from P3. Clinical patency alone is not an adequate proxy, because fabric or interface failure can occur without an immediate patency signal under the LBV combination of focal crush, compression-recovery cycling, and multi-axial bending (Eadie et al., 2014; Lin et al., 2016). What is genuinely optional, and therefore assigned to P3, is an added biological or surface function: a drug, an active antithrombotic or antiproliferative coating, or another functional surface. Such layers cannot be transferred directly from arterial drug-elution experience, because the venous low-pressure, low-velocity, high-access-flow, and high-reintervention setting alters release kinetics, healing, thrombosis, and coating-durability risk. They are considered only after P1 and P2 evaluation of the base platform, and any layer that alters platform mechanics or the flow field requires repeat evaluation (Williams et al., 2023; Eadie et al., 2014; Lin et al., 2016).

### 6.7 Synthesis of P1/P2/P3 priorities and unresolved design gaps

Current venous stent limitations in the LBV should be converted into reportable, reproducible, and comparable engineering metrics. To prevent Figure 7 and Table 5 from being read as equal-weight lists, the framework distinguishes two required stages from one optional stage, the prefix P denoting a validation priority stage. P1 and P2 are the required mechanical and hemodynamic evaluations of the base platform, itemized in Table 5 and assessed against a prespecified indication-relevant comparator panel under identical conditions; P3 is an optional biological or surface optimization considered only after P1/P2 feasibility. A construct completing P1 and P2 is described as a P1/P2-screened platform.

**TABLE 5:** Staged P1/P2/P3 validation priorities for LBV-oriented stent concepts. P1 is the required mechanical, structural, fatigue, and deployment evaluation of the base platform; P2 is the required anatomy-specific hemodynamic evaluation of the same platform using computational and, where feasible, experimental methods; P3 is an optional additional biological or surface optimization considered after P1/P2 feasibility. Baseline thrombogenicity, hemocompatibility, and tissue compatibility are mandatory cross-cutting base-platform safety requirements assessed alongside the staged framework. Structural integrity and, for covered constructs, cover–frame integrity and covering fatigue remain required P1 components. This is a research framework, not a pass-fail standard, a regulatory requirement, or a product ranking. Abbreviations: COF, chronic outward force; CFD/FSI, computational fluid dynamics/fluid–structure interaction; ISR, in-stent restenosis; LBV, left brachiocephalic vein; OSI, oscillatory shear index; RRF, radial resistive force; RRT, relative residence time; SVC, superior vena cava; TAWSS, time-averaged wall shear stress; WSS, wall shear stress.

Priority	Design focus	LBV-specific rationale	Minimum validation path
P1 (required): mechanical, structural, fatigue, and deployment evaluation of the base platform	Scaffold mechanics for uncovered, hybrid, or covered constructs; focal unidirectional compression resistance and post-unloading recovery; RRF/COF balance; bending and torsion fatigue; for nitinol constructs, the mean and alternating components of maximum principal strain at the sternum–aortic contact and other critical locations; axial compliance or incremental-stiffness transition at the proximal and distal stent edges; structural integrity; branch preservation; low foreshortening and deployment precision; re-entry feasibility; and, for a covered construct, cover-frame integrity and covering fatigue	Addresses the core LBV phenotype: sternum–aortic compression, long transverse angulation, short landing zones at the lateral venous angle or medial brachiocephalic–SVC confluence, and future access-resource preservation	LBV-specific focal compression, compression-recovery, and multi-axial fatigue tests with explicitly defined diameter reduction; nonlinear finite-element strain assessment and material-specific constant-life data for nitinol constructs; axial compliance or incremental-stiffness profiling across each landing-zone transition; covering-fatigue and cover-frame integrity tests for covered constructs; prespecified indication-relevant comparator panel or matched prototypes under identical conditions; endpoint-specific margins and 95% CIs; deployment-error, foreshortening, branch-access/re-entry, and residual-diameter assessments; report the multi-objective trade-off profile
P2 (required): anatomy-specific hemodynamic evaluation of the base platform	Patient-specific hemodynamic compatibility; matched virtual-repair and reference-stent comparisons; non-physiologic shear; edge recirculation and washout; branch-flow and pressure-loss preservation; CFD/FSI or transparent-flow models	Pre-intervention stenosis, stent-induced lumen and edge geometry, access flow, and branch inflows create non-comparable flow states; low WSS alone is not a monotonic optimization target.	Virtual deployment under identical target-lumen and boundary conditions; smooth-lumen and reference-stent comparators; report TAWSS, OSI, RRT, WSS gradient, recirculation/washout, branch flow, and pressure loss with experimental validation and sensitivity/uncertainty analysis
P3 (optional): added biological or surface layer considered after P1/P2 feasibility	An added drug, active antithrombotic or antiproliferative coating, or other functional surface; release behavior, coating integrity, and its incremental effect on endothelial healing and thromboresistance. Baseline hemocompatibility, thrombogenicity, and tissue compatibility are not part of this optional stage; they are mandatory cross-cutting safety requirements. Covering integrity remains a required P1 attribute for covered constructs	May further address thrombus organization, edge stenosis, and intimal hyperplasia, but cannot compensate for a base platform that fails P1 mechanics or P2 flow compatibility, and is not required for a P1/P2-screened uncoated platform to proceed	Venous-flow release kinetics; platelet, coagulation, and complement assays; endothelial and smooth-muscle-cell compatibility; coating integrity after crimping, deployment, bending, and compression; *ex vivo* or animal flow-loop validation, benchmarked against the uncoated P1/P2-screened platform

These considerations can be summarized as three engineering and biomaterial gaps. First, current mechanical tests are based largely on the iliofemoral venous environment and do not reproduce unidirectional LBV compression, multi-axial bending, end-anchoring coupling, or short junctional landing zones. Second, a single uniform topology is unlikely to satisfy the zoned mechanical needs of the compression segment, curved segment, and confluence simultaneously. Third, branch preservation, low foreshortening, re-entry feasibility, covering integrity, coating durability, hemocompatibility, and long-term vascular-resource protection still lack systematic device solutions (Lok et al., 2020; Dabir et al., 2018; Liao et al., 2024; Ginsburg et al., 2015; Xu et al., 2021; Theodoridis et al., 2024; Smith et al., 2025; Lim and Black, 2022; Schwein et al., 2018; Maleckis et al., 2018; Maleckis et al., 2017; Cheng et al., 2020; Hu et al., 2024; Eadie et al., 2014; Lin et al., 2016). Figure 7 and Table 5 assign these gaps to the stages defined above; expanded metrics are given in Supplementary Table S4 and terminology in Supplementary Table S3.

### 6.8 Stage-gate logic: entry, decision, and failure criteria for P1/P2/P3

Table 5 organizes candidate metrics by stage; Table 6 specifies entry conditions, comparator strategy, endpoint-specific decision rules, and failure handling. P1 and P2 are required gates for the base platform, whereas P3 is an optional branch applying only when an added drug, active coating, or functional surface is included. P1 does not use a hypothetical “best non-LBV benchmark”: before testing, investigators should identify one or more indication-relevant reference devices or matched internal prototypes, justify their selection, and lock device size, length, deployment and oversizing, fixture, temperature, curvature, and end constraints. Safety-critical endpoints use absolute criteria, continuous endpoints separately justified margins with confidence intervals, and Pareto reporting cannot rescue a safety-critical failure. Because LBV-specific absolute thresholds are not established, the framework prescribes neither universal numeric margins nor a universally superior comparator. P2 retains the matched post-repair comparator logic described above, and longitudinal clinical imaging sits after these gates as a correlation step rather than a P2 release criterion.

**TABLE 6:** Compact stage-gate summary for the P1/P2/P3 validation roadmap. P1 and P2 are required base-platform gates; P3 is optional only for an added drug, active coating, or functional surface. The full entry criteria, comparator definitions, endpoint-specific decision rules, and failure handling are provided in Supplementary Table S7. Completion of P1/P2 screening does not imply clinical validation, efficacy, or superiority.

Stage	Entry/focus	Core test and readouts	Gate criterion to advance	Failure handling
P1 (required)	Base-platform mechanical, structural, fatigue, and deployment evaluation	Focal crush and recovery; bending/torsion fatigue; foreshortening; deployment and branch preservation; matched indication-relevant comparators	Absolute safety criteria pass and each continuous endpoint meets its justified margin with 95% CI.	Redesign the failed segment, material, or deployment interaction and repeat the locked comparison; a safety-critical P1 failure stops progression
P2 (required)	Anatomy-specific hemodynamic evaluation of the same P1-screened platform	Patient-specific CFD/FSI or flow-loop testing; smooth-lumen and reference-stent comparators; WSS/OSI/RRT, recirculation, branch flow, and pressure loss	Prespecified disturbed-flow burden is non-inferior or improved versus the reference envelope, with robust sensitivity/uncertainty results	Revise geometry, edge transition, apposition, landing zone, or deployment and repeat P2; P3 cannot rescue an unfavorable base-platform flow field
P3 (optional)	Added drug, active coating, or functional surface after P1/P2 feasibility	Release kinetics; coating integrity; platelet/coagulation/complement assays; endothelial and smooth-muscle-cell compatibility versus the uncoated P1/P2 platform	No degradation of P1/P2 performance and acceptable incremental hemocompatibility or surface-function results; altered mechanics/flow trigger repeat testing	Revert to the uncoated P1/P2-screened platform and treat the added layer as a separate iteration
Translational endpoint	Integrated clinical translation after the applicable gates	Prospective imaging–patency registry with standardized lesion, device, branch, reintervention, and access-survival endpoints	Correlation of prespecified P1/P2 outputs with residual stenosis, deformation, patency, and reintervention	No universal LBV threshold or device-ranking claim is made; report uncertainty and evidence gaps

## 7 Discussion

The principal synthesis is that HD-related LBV stenosis is an anatomy-specific device environment rather than a smaller instance of generic CVS. Three interpretations follow. First, retrosternal focal compression, multi-axial curvature, catheter or device exposure, high-flow access return, and junction constraints may coexist, yet none is assumed to be necessary or dominant in every patient. Second, recurrent failure after PTA, BMS, or SG identifies unresolved mechanical, deployment, flow, and tissue-response boundaries, but the heterogeneous evidence supports neither device ranking nor routine primary stenting. Third, the most defensible near-term development sequence evaluates the base platform mechanically and for deployment (P1), then hemodynamically (P2), with an optional biological or surface layer (P3) only afterwards.

Clinically, this framework shifts emphasis from stenosis percentage or device category toward symptoms and access dysfunction, lesion mechanism, segment geometry, branch preservation, and reintervention feasibility. After repeated endovascular failure or exhaustion of conventional upper-limb access, right atrial bypass, Hemodialysis Reliable Outflow (HeRO) grafts, lower-limb access, and other unconventional options remain salvage pathways for highly selected patients (Paik et al., 2024; Pride et al., 2024; Xu et al., 2024), reinforcing the importance of vascular-resource preservation in initial device design. Because direct prospective LBV evidence remains sparse, candidate platforms should be compared through endpoint-defined mechanical survival, deployment accuracy, flow compatibility, branch/access preservation, and reintervention durability, validated through standardized imaging–patency registries rather than extrapolation from iliofemoral or mixed-CVS cohorts.

### 7.1 Future validation and research priorities

Future validation should integrate clinical, engineering, biomaterial, and computational pathways around the staged roadmap in Tables 5 and 6. The P1 bench programme must be run on prespecified indication-relevant comparators with endpoint-specific margins, confidence intervals, and explicit trade-off reporting, and must include cover-frame integrity and covering fatigue for covered constructs. The P2 flow programme must compare candidate deployment with both a flow-matched smooth-lumen repair and a clinically relevant reference stent under identical target-lumen and boundary conditions, prespecify a primary disturbed-flow endpoint, and test robustness to geometry, inflow, outlet, respiratory, wall, rheology, mesh, and stent-representation uncertainty. An optional P3 branch applies only to a drug, coating, or functional surface, linking layer integrity and release behavior to thrombus and ISR localization, hemocompatibility, healing, and access preservation. Model credibility should be documented with the applicable solid-mechanics, CFD, and risk-informed medical-device verification and validation frameworks (American Society of Mechanical Engineers, 2009; American Society of Mechanical Engineers, 2018; American Society of Mechanical Engineers, 2019).

Illustrative validation sequence. Consider a maintenance HD patient with a left upper-limb AVF, no history of central venous catheterization, and LBV stenosis dominated by sternum–aortic compression, in whom CTV shows a narrow retrosternal corridor, multi-axial angulation, and a landing zone shorter than the intended device length adjacent to the medial brachiocephalic–SVC confluence. All numeric values below are illustrative reporting points, not validated LBV thresholds; corridor width and landing-zone length must be measured in the individual patient.

P1 would test whether a candidate concept maintains geometry under these constraints. Testing would include flat-plate and wedge-based unidirectional compression at 10%, 25%, 50%, and 65% diameter reduction, equivalent to 90%, 75%, 50%, and 35% residual nominal diameter and reported as 100 × (*D*_0_ − *D*)/*D*_0_, together with chronic compression-hold, recovery after unloading, and repeated external-compression cycling (Lim and Black, 2022; Schwein et al., 2018; Maleckis et al., 2018; Maleckis et al., 2017). The lowest point is included because the reported corridor widths imply compression beyond the 50% level at which published venous-stent comparisons stop. Multi-axial bending fatigue would reproduce the CTV-derived curvature envelope and superimpose the two load sources that generic protocols omit: respiratory cycling, since the sternum–aortic distance narrows and widens with each breath, and upper-limb-motion loading transmitted from the AVF-bearing arm toward the lateral venous angle, by analogy with hip-motion effects documented for iliofemoral venous stents (Cheng et al., 2020). Neither has an established LBV-specific amplitude, so both are test inputs to be measured (Supplementary Table S5), and for a covered construct cover-frame integrity and covering fatigue would be assessed under the same loading (Eadie et al., 2014; Lin et al., 2016). Low-foreshortening and deployment-accuracy tests would use a short-landing-zone phantom, and branch-access preservation would be tested by re-crossing, fenestration, or branch-orifice access tasks after simulated deployment (Hu et al., 2024).

P2 would retain the pre-intervention stenotic model to describe the disease-state jet, pressure loss, and flow division, but would base acceptance on the two post-repair controls defined above under identical time-resolved boundary conditions, with the assumptions in Supplementary Table S4 prespecified and examined in sensitivity and uncertainty analyses (McCarthy et al., 2025). Advancement would require non-inferior or improved disturbed-flow burden relative to the reference stent, preserved branch-flow distribution, acceptable pressure loss, and no new persistent edge recirculation or high-gradient jet. For H2, an early post-implant flow map obtained before appreciable ISR would be spatially registered with later imaging, since a late model that already contains ISR cannot by itself establish causal direction (Ninno et al., 2025; Torikai et al., 2026; Zhang et al., 2026).

An optional P3 layer, considered only after P1 and P2 are acceptable, would require separate testing of coating integrity, release kinetics, incremental hemocompatibility, and cell compatibility against the uncoated platform. The clinical bridge would be a prospective imaging–patency registry linking these outputs to residual stenosis, branch patency, stent deformation, primary patency, reintervention, and access survival. This example is an illustrative framework, not a validated regulatory or clinical testing pathway.

### 7.2 Limitations

This Review has five major limitations. First, it is a critical narrative rather than a preregistered systematic review, so literature-selection bias may remain. Second, direct LBV clinical evidence is limited, several anchoring anatomical datasets are small single-center cohorts, and many studies pool brachiocephalic, subclavian, and SVC lesions with inconsistent patency definitions and follow-up. Third, the stent-design, hemodynamic, and candidate biological-hypothesis sections rely partly on indirect extrapolation from iliocaval, femoropopliteal, SVC, and AVF/AVG settings, which differ from the LBV in loading geometry, junction constraints, and flow state. Fourth, the mechanical test standards are cited for scope and method rather than acceptance criteria, none defines LBV-specific loading, amplitudes, cycle counts, or acceptance thresholds, and the loading spectrum given above is an arithmetic projection rather than a measured LBV load spectrum. Fifth, the Review emphasizes anatomical and mechanical drivers but has not systematically weighed competing explanations that could inflate the apparent LBV-specificity of the phenotypes described. Systemic factors such as chronic kidney disease-mineral and bone disorder and uremic vascular calcification may affect central veins regardless of segment-specific anatomy. Operator- and technique-dependent variables such as balloon sizing, inflation pressure, and stent oversizing contribute to restenosis independently of anatomical compression. Because LBV lesions tend to produce conspicuous symptoms such as facial or periorbital swelling and intracranial venous reflux, they may be preferentially imaged and studied, inflating their apparent prevalence relative to less symptomatic segments. These explanations have not been excluded; future LBV-specific studies should report calcification burden, technique parameters, and referral pathway alongside anatomical and mechanical variables. The Review therefore proposes a problem definition and a candidate validation framework, not proof of efficacy for any device platform and not a clinical recommendation.

## 8 Conclusion

HD-related LBV stenosis should be understood as an anatomy-specific functional-biomaterial and venous-device problem, in which retrosternal compression, multi-axial angulation, catheter/device injury, high-flow access return, and lateral-venous-angle and medial brachiocephalic–SVC anatomy jointly shape stent failure. The central contribution of this Review is not to recommend a device or treatment pathway, but to organize this problem into a translational sequence: anatomy-specific exposure conditions, recurrent failure phenotypes, biomaterial and venous-device constraints, and staged validation requirements.

Current PTA, BMS, and SG can relieve symptoms and maintain access function, but their long-term benefit remains limited by elastic recoil, ISR, edge stenosis, branch coverage, and repeated reintervention (Andrawos et al., 2021; Agarwal, 2015; Liu et al., 2024; Chawla et al., 2025). Future LBV-oriented concepts are therefore evaluated here through the staged P1/P2/P3 roadmap, in which covering and structural integrity remain required base-platform attributes and baseline hemocompatibility, thrombogenicity, and tissue compatibility remain mandatory cross-cutting safety requirements rather than optional additions. Uncovered scaffolds, covered constructs, and future functionalized platforms should be compared by the endpoints each is designed to satisfy, under identical test conditions and against indication-relevant comparators, rather than by category labels alone.
