# Supplementary material for: Stent biomechanics in hemodialysis-related left brachiocephalic vein stenosis: from site-specific loading to a staged device-validation framework
Source: Front Bioeng Biotechnol. 2026 Sep 30;14:1961298. doi: 10.3389/fbioe.2026.1961298 (PMC13662427; doi:10.3389/fbioe.2026.1961298)
Supplement: Supplementary file 1 [file DataSheet1.pdf]

All citations in this supplementary file correspond to the reference list of the main manuscript.

This supplementary material accompanies the main manuscript and contains Supplementary Box S1 and Supplementary Tables S1-S7, presented in numerical order. Its purpose is to preserve traceable mechanistic details, device-level evidence, search transparency, standardized terminology, and the distinction between observed phenomena, extrapolated mechanisms, and downstream hypotheses; it does not add independent clinical recommendations.

Supplementary Box S1. Hemodynamic mechanisms and downstream biological candidate pathways

Unidirectional pulsatile shear versus oscillatory shear: In vitro cellular studies suggest that unidirectional pulsatile WSS can upregulate KLF2 and support an endothelial-protective phenotype, whereas oscillatory WSS may maintain a cobblestone-like morphology, reduce protective transcriptional programs, and increase pro-inflammatory/pro-proliferative signals such as MCP-1 and IL-8 (Franzoni et al., 2016).

CFD/FSI extrapolation: AVF/AVG, venous-stent, and bifurcation/curvature models identify low or oscillatory shear, steep WSS gradients, persistent recirculation, and prolonged residence as candidate lesion-associated patterns. These metrics do not establish a single causal direction: an established stenosis or ISR can itself alter the local flow field and reduce or redistribute high- and low-WSS regions (Ene-Iordache and Remuzzi, 2012; Torikai et al., 2026; Zhang et al., 2026). LBV validation therefore requires matched post-repair comparators and an early post-implant flow map obtained before appreciable ISR, followed by later spatial ISR registration.

ROS/NF-κB axis: Disturbed flow and cyclic external compression may induce ROS generation and activate NF-κB-related inflammatory cytokines, chemokines, and adhesion molecules. The direct role of this axis in LBV in-stent restenosis tissue has not been validated (Helmcke et al., 2009; Chandel et al., 2000; Reynaert et al., 2006).

Downstream biological candidate pathways: Piezo1 and ROS/NF-κB may be candidate links between mechanical stimulation, inflammation, migration, matrix remodeling, and proliferation. These details are retained in the supplement to keep the main text focused on measurable mechanical, hemodynamic, and branch-preservation validation domains (Shinge et al., 2021; Zeng et al., 2025; Shinge et al., 2022).

Evidence boundary for Supplementary Box S1: The hemodynamic propositions are supported primarily by Analogous engineering evidence, and the downstream pathways are Mechanistic hypotheses; S1 contains no Direct LBV stent-tissue evidence. The proposition that these pathways connect abnormal mechanical environments with inflammation, thrombus organization, and intimal hyperplasia requires LBV stent histopathology, spatial omics, dynamic compression models, and imaging–CFD/FSI–pathology correlation before causal interpretation.

Supplementary Table S1. Device-level bench and clinical evidence supporting main-text design implications

Device / source	Device-level evidence (with citation)	Design implication retained	Evidence boundary and evidence proximity	Regulatory/indication note relevant to the LBV (market-specific)
Venous-stent bench comparison, Venovo (Dabir et al., 2018)	In a single bench study applying one protocol to seven venous stents,	Within-study ordering obtained in an iliocaval parallel-plate setting	Bench evidence only; does not represent LBV clinical efficacy.	

Device / source	Device-level evidence (with citation)	Design implication retained	Evidence boundary and evidence proximity	Regulatory/indication note relevant to the LBV (market-specific)
	Venovo showed a lower maximum radial resistive force than several comparators, whereas its chronic outward force was highest at 90% and 80%residual nominal diameter. These protocol- and fixture-dependent findings should be interpreted only within the study and do not establish overall device superiority.	cannot be extrapolated to the LBV; focal unidirectional compression testing at defined residual nominal diameters is needed.	Evidence proximity: Analogous engineering.	U.S. PMA P180037: indicated for symptomatic iliofemoral venous-outflow obstruction. LBV use is outside this U.S. labeled anatomical indication (U.S. Food and Drug Administration, 2019).
Crush-versus-radial ordering across devices (Dabir et al., 2018)	In the same bench study, the Wallstent showed the highest crush resistance between 90% and 60% of nominal diameter, whereas Sinus-Obliquus showed both the highest radial resistive force and the highest crush resistance at 50% of nominal diameter.	Crush resistance and radial resistive force are related but non-redundant descriptors, and their relative ordering is compression-level dependent. This is the empirical basis for H1 and the reason both should be reported across a defined range of residual nominal diameter rather than at a single point.	Single iliocaval bench study; the two quantities were obtained on different apparatus, so absolute magnitudes must not be compared as a ratio. Evidence proximity: Analogous engineering.	See the product-specific U.S. indication for Wallstent below. Sinus-Obliquus is included only as a bench comparator.
Wallstent end-embedding effect (Dabir et al., 2018)	Radial resistance was low when the stent was unconstrained and increased markedly when the ends were constrained.	In vivo performance depends on stent-vessel wall boundary coupling; end anchoring zones and the central functional segment should be zoned.	Bench simulation; requires validation in LBV anatomy under dynamic external compression. Evidence proximity: Analogous engineering.	Specified Wallstent configurations are indicated in the United States for improving central venous luminal diameter after unsuccessful angioplasty in chronic-hemodialysis patients with innominate or subclavian venous-outflow stenosis; only the listed product codes and label conditions apply (Boston Scientific, n.d.).
Venovo thoracic central venous experience (Maleux et al., 2024)	SVC obstruction cohorts showed relatively high short- to mid-term primary patency, but malignant lesions predominated.	Deployment strategy, anchoring zones, and oversizing strategy may inform LBV planning.	Malignant-obstruction cohort; cannot be extrapolated to benign dialysis-related LBV disease. Evidence proximity: Analogous engineering.	U.S. PMA P180037 concerns symptomatic iliofemoral venous-outflow obstruction; thoracic central-venous or LBV use is outside that labeled anatomical indication (U.S. Food and Drug Administration, 2019).
Abre dialysis CVO experience (Aronhime et al., 2024)	A small HD CVO series reported high technical success with no observed stent fracture across the	A flexible open-cell structure is compatible with, but is not demonstrated by these data to provide, adequate fatigue	Single-center, small sample, limited follow-up; an absence of observed fracture in a small short-follow-up series has low statistical power to	U.S. PMA P200026: indicated for

Device / source	Device-level evidence (with citation)	Design implication retained	Evidence boundary and evidence proximity	Regulatory/indication note relevant to the LBV (market-specific)
	thoracic inlet.	performance in high-stress segments.	exclude fatigue failure, and geometric topology alone cannot solve ISR. Evidence proximity: Adjacent HD-CVS.	use in the iliofemoral veins for symptomatic venous-outflow obstruction; LBV use is outside that labeled anatomical indication (U.S. Food and Drug Administration, 2020a).
Zilver Vena and branch-preservation concepts (Comerota et al., 2024; Theodoridis et al., 2024)	Iliofemoral experience suggests that low foreshortening and precise deployment help avoid contralateral venous coverage; a Y-shaped stent case suggests possible functional preservation.	The lateral venous angle and medial brachiocephalic–SVC confluence require separate deployment models, with junction-specific tests of foreshortening, branch preservation, and re-entry feasibility.	Non-LBV-specific evidence or single-case experience; systematic structural and long-term patency validation is required. Evidence proximity: Analogous engineering + Direct LBV case evidence.	Zilver Vena, U.S. PMA P200023: indicated for improving luminal diameter in the iliofemoral veins for symptomatic iliofemoral venous-outflow obstruction (U.S. Food and Drug Administration, 2020b).

Supplementary Table S2. Search domains, database-adapted search strings, and evidence-use boundaries

This table documents how literature was identified for the structured critical narrative Review. It reports the databases, search update date, domain-specific search concepts, database-adapted strings, and limits placed on inference. It is not a systematic-review protocol or study-selection log: database yields were not used as denominators, stage-specific screening counts were not compiled, and no PRISMA flow diagram is presented. Database searches were updated through May 20, 2026; targeted citation surveillance through July 20, 2026 identified newly published studies that directly changed interpretation of H2 and the P2 comparator logic.

S2-1. Database-adapted search strings and source roles

Evidence domain	PubMed/MEDLINE search string	Web of Science adaptation and use	Embase adaptation and use	Google Scholar and citation-tracking use
Direct clinical and anatomical evidence for LBV/brachiocephalic vein	(("left brachiocephalic vein"[tiab] OR "left innominate vein"[tiab] OR "brachiocephalic vein"[tiab] OR "innominate vein"[tiab]) AND (stenosis[tiab] OR obstruction[tiab] OR occlusion[tiab] OR compression[tiab]) AND (hemodialysis[tiab] OR dialysis[tiab] OR "vascular access"[tiab] OR "arteriovenous fistula"[tiab] OR	TS=(("left brachiocephalic vein" OR "left innominate vein" OR "brachiocephalic vein" OR "innominate vein") AND (stenosis OR obstruction OR occlusion OR compression) AND (hemodialysis OR dialysis OR "vascular access" OR "arteriovenous fistula" OR	("left brachiocephalic vein":ti,ab OR "left innominate vein":ti,ab OR "brachiocephalic vein":ti,ab OR "innominate vein":ti,ab) AND (stenosis:ti,ab OR obstruction:ti,ab OR occlusion:ti,ab OR compression:ti,ab) AND (hemodialysis:ti,ab OR dialysis:ti,ab OR "vascular access":ti,ab OR	"left brachiocephalic vein" hemodialysis stenosis stent; "innominate vein" compression dialysis access; key cited-reference chasing.

Evidence domain	PubMed/MEDLINE search string	Web of Science adaptation and use	Embase adaptation and use	Google Scholar and citation-tracking use
	"arteriovenous graft"[tiab] OR stent[tiab] OR angioplasty[tiab])).	"arteriovenous graft" OR stent OR angioplasty)). Field: Topic/TS; used for coverage checking and unique candidate confirmation.	"arteriovenous fistula":ti,ab OR "arteriovenous graft":ti,ab OR stent:ti,ab OR angioplasty:ti,ab). Field: title/abstract; used for coverage checking.	
General HD-related CVS evidence, guidelines, and treatment outcomes	(("central venous stenosis"[tiab] OR "central vein stenosis"[tiab] OR "central venous occlusion"[tiab] OR "central venous disease"[tiab]) AND (hemodialysis[tiab] OR dialysis[tiab] OR "vascular access"[tiab] OR "arteriovenous fistula"[tiab] OR "arteriovenous graft"[tiab]) AND (angioplasty[tiab] OR stent[tiab] OR "stent graft"[tiab] OR patency[tiab] OR guideline[tiab] OR review[pt])).	TS=(("central venous stenosis" OR "central vein stenosis" OR "central venous occlusion" OR "central venous disease") AND (hemodialysis OR dialysis OR "vascular access" OR "arteriovenous fistula" OR "arteriovenous graft") AND (angioplasty OR stent OR "stent graft" OR patency OR guideline OR review)). Field: Topic/TS; used for coverage checking and unique candidate confirmation.	("central venous stenosis":ti,ab OR "central vein stenosis":ti,ab OR "central venous occlusion":ti,ab OR "central venous disease":ti,ab) AND (hemodialysis:ti,ab OR dialysis:ti,ab OR "vascular access":ti,ab OR "arteriovenous fistula":ti,ab OR "arteriovenous graft":ti,ab) AND (angioplasty:ti,ab OR stent:ti,ab OR "stent graft":ti,ab OR patency:ti,ab OR guideline:ti,ab OR review:ti,ab). Field: title/abstract; used for coverage checking.	central venous stenosis hemodialysis KDOQI stent graft; dialysis access central venous occlusion patency; guideline/systematic review citation chasing.
ISR, hemodynamics, and mechanobiology evidence	((("in-stent restenosis"[tiab] OR "neointimal hyperplasia"[tiab] OR thrombosis[tiab]) AND ("venous stent"[tiab] OR "stent graft"[tiab] OR "vascular access"[tiab] OR "arteriovenous fistula"[tiab] OR "arteriovenous graft"[tiab] OR hemodialysis[tiab] OR dialysis[tiab])) OR (("wall shear stress"[tiab] OR hemodynamics[tiab] OR "oscillatory shear"[tiab] OR "relative residence time"[tiab] OR Piezo1[tiab] OR NLRP3[tiab] OR "NF-kappaB"[tiab]) AND ("vascular access"[tiab] OR "arteriovenous fistula"[tiab] OR "arteriovenous graft"[tiab] OR "venous stent"[tiab] OR hemodialysis[tiab] OR dialysis[tiab]))). This deliberately broad string was used for hypothesis discovery; resulting sources were interpreted only as mechanistic or hemodynamic support.	TS=((("in-stent restenosis" OR "neointimal hyperplasia" OR thrombosis) AND ("venous stent" OR "stent graft" OR "vascular access" OR "arteriovenous fistula" OR "arteriovenous graft" OR hemodialysis OR dialysis)) OR (("wall shear stress" OR hemodynamics OR "oscillatory shear" OR "relative residence time" OR Piezo1 OR NLRP3 OR "NF-kappaB") AND ("vascular access" OR "arteriovenous fistula" OR "arteriovenous graft" OR "venous stent" OR hemodialysis OR dialysis))). Field: Topic/TS; used for coverage checking and unique candidate confirmation.	(("in-stent restenosis":ti,ab OR "neointimal hyperplasia":ti,ab OR thrombosis:ti,ab) AND ("venous stent":ti,ab OR "stent graft":ti,ab OR "vascular access":ti,ab OR "arteriovenous fistula":ti,ab OR "arteriovenous graft":ti,ab OR hemodialysis:ti,ab OR dialysis:ti,ab)) OR (("wall shear stress":ti,ab OR hemodynamics:ti,ab OR "oscillatory shear":ti,ab OR "relative residence time":ti,ab OR Piezo1:ti,ab OR NLRP3:ti,ab OR "NF-kappaB":ti,ab) AND ("vascular access":ti,ab OR "arteriovenous fistula":ti,ab OR "arteriovenous graft":ti,ab OR "venous stent":ti,ab OR hemodialysis:ti,ab OR dialysis:ti,ab)). Field: title/abstract; used for coverage checking.	"in-stent restenosis" venous stent hemodialysis; "wall shear stress" arteriovenous fistula stenosis; Piezo1 vascular access restenosis; mechanism papers used only as hypothesis-level support.
Venous stent engineering and device design evidence	(("venous stent"[tiab] OR Wallstent[tiab] OR Venovo[tiab] OR Abre[tiab] OR "Zilver Vena"[tiab] OR "Sinus Venous"[tiab]) AND ("radial resistive force"[tiab] OR "chronic outward	TS=(("venous stent" OR Wallstent OR Venovo OR Abre OR "Zilver Vena" OR "Sinus Venous") AND ("radial resistive force" OR "chronic outward force" OR "crush resistance" OR	("venous stent":ti,ab OR Wallstent:ti,ab OR Venovo:ti,ab OR Abre:ti,ab OR "Zilver Vena":ti,ab OR "Sinus Venous":ti,ab) AND ("radial resistive force":ti,ab OR "chronic outward	"Venovo" radial resistive force; "Abre" venous stent fatigue; "Wallstent" chronic outward force; device names plus bench testing/reference

Evidence domain	PubMed/MEDLINE search string	Web of Science adaptation and use	Embase adaptation and use	Google Scholar and citation-tracking use
	force"[tiab] OR "crush resistance"[tiab] OR flexibility[tiab] OR conformability[tiab] OR "bench testing"[tiab] OR fatigue[tiab] OR "stent design"[tiab])).	flexibility OR conformability OR "bench testing" OR fatigue OR "stent design")). Field: Topic/TS; used for coverage checking and unique candidate confirmation.	force":ti,ab OR "crush resistance":ti,ab OR flexibility:ti,ab OR conformability:ti,ab OR "bench testing":ti,ab OR fatigue:ti,ab OR "stent design":ti,ab). Field: title/abstract; used for coverage checking.	chasing.

S2-2. Evidence-use boundaries

Direct LBV/brachiocephalic evidence was preferentially used for site-specific anatomical and clinical claims. General HD-CVS evidence was used for treatment boundaries and access-preservation principles. Hemodynamic, mechanobiological, and non-LBV device evidence was used to generate candidate mechanisms, design requirements, or validation questions and was not treated as proof of LBV causality or device superiority. Case reports and small series were used only to illustrate phenotype or feasibility. Sources were not carried forward when they lacked interpretable relevance to dialysis access, thoracic central venous anatomy, venous-stent failure, or device design, or when only non-verifiable product publicity was available.

Supplementary Table S3. Abbreviations and terms

Abbreviation	Full term	Definition/use in this review
LBV	left brachiocephalic vein	Primary anatomical segment addressed in this review
RBV	right brachiocephalic vein	Comparator anatomical segment. Note: the same vessel is labelled RBCV in Figure 4 of the main manuscript; RBV and RBCV are used interchangeably and denote the right brachiocephalic vein.
CVS	central venous stenosis	Central venous narrowing
CVO	central venous occlusion	Central venous occlusion
CVD	central venous disease	General central venous disease
HD	hemodialysis	Hemodialysis
MHD	maintenance hemodialysis	Maintenance hemodialysis
AVF	arteriovenous fistula	Dialysis arteriovenous fistula
AVG	arteriovenous graft	Dialysis arteriovenous graft

Abbreviation	Full term	Definition/use in this review
PTA	percutaneous transluminal angioplasty	Balloon angioplasty
BMS	bare-metal stent	Uncovered metallic stent
SG	stent graft	Covered stent/stent graft
ISR	in-stent restenosis	Restenosis within a stent
WSS	wall shear stress	Wall shear stress
OSI	oscillatory shear index	Oscillatory shear index
RRT	relative residence time	Relative residence time
TAWSS	time-averaged wall shear stress	Wall shear stress averaged over the cardiac cycle
CFD	computational fluid dynamics	Computational fluid-dynamics modeling
FSI	fluid-structure interaction	Fluid-structure interaction modeling
FEM/FEA	finite-element method / finite-element analysis	Computational structural modeling of stent mechanics
RRF	radial resistive force	Radial resistive force
COF	chronic outward force	Chronic outward force
SVC	superior vena cava	Superior vena cava
PICC	peripherally inserted central catheter	Peripherally inserted central catheter
KDOQI	Kidney Disease Outcomes Quality Initiative	Refers mainly to KDOQI vascular access guideline recommendations
ROS	reactive oxygen species	Reactive oxygen species
NF-κB	nuclear factor κB	Nuclear factor κB pathway
NLRP3	NLR family pyrin domain containing 3	NLRP3 inflammasome-related pathway
FAK	focal adhesion kinase	Focal adhesion kinase
KLF2	Kruppel-like factor 2	Endothelial protective transcription factor
HeRO	Hemodialysis Reliable Outflow	HeRO graft/outflow system

Abbreviation	Full term	Definition/use in this review
CTV	computed tomography venography	Computed tomography venography
MDT	multidisciplinary team	Multidisciplinary team
PIV	particle image velocimetry	Experimental flow-loop validation of CFD/FSI predictions
4D-flow MRI	four-dimensional flow magnetic resonance imaging	Time-resolved in vivo flow measurement where available

Supplementary Table S4. Full candidate design metrics and validation framework for LBV-oriented stent concepts

This table is presented in the supplementary material because of its width, which would compress readability in the main text. It should be interpreted as a candidate validation framework, not as a product recommendation, qualification threshold, validated design rule, or clinical algorithm. The mechanical and engineering categories in this table are additionally supported by focused pre-submission engineering references on venous-stent mechanics, nitinol deformation, bifurcation hemodynamics, and covered-stent-graft material fatigue (Lim and Black, 2022; Schwein et al., 2018; Maleckis et al., 2018; Maleckis et al., 2017; Cheng et al., 2020; Hu et al., 2024; Eadie et al., 2014; Lin et al., 2016).

Design dimension	Priority and LBV-specific rationale	LBV pathological/engineering scenario	Candidate quantitative metrics	Suggested testing/data source	Reporting format and validation status	Risk if ignored
Unidirectional compression resistance and radial support	P1; LBV-specific. The key difference is sternum–aortic arch unidirectional compression rather than simple circumferential recoil.	Local compression between sternum and aortic arch; post-PTA recoil; LBV lesions with prominent extrinsic compression or high compression risk.	Focal-crush load–displacement curve at explicitly defined diameter reduction; residual diameter/area and ellipticity; recovery after hold; RRF and COF reported separately; bending stiffness; post-fatigue change; fracture or permanent deformation.	Flat-plate and wedge compression at 10%, 25%, 50%, and 65% diameter reduction (90%, 75%, 50%, and 35% residual nominal diameter), with imaging-derived primary range; chronic hold/recovery; repeated compression plus bending/torsion. Prespecified comparator panel tested side by side at matched size, length, oversizing, temperature, fixture, curvature, end constraints, and final	Define diameter reduction as 100 × (D0 − D)/D0. Lock comparators, endpoints, absolute safety criteria, performance-specific superiority/equivalence/non-inferiority margins, sample-size rationale, and analysis before testing. Report independent units, mean/dispersion and 95% CIs, full curves, failures, and a multi-objective/Pareto trade-off display. No universal LBV margin or threshold is established.	False device ranking from unmatched fixtures or post hoc comparator selection; ambiguous compression percentages; hidden trade-offs between support, COF, flexibility, foreshortening, and branch access; fracture, permanent collapse, wall overstimulation, or branch loss.

Design dimension	Priority and LBV-specific rationale	LBV pathological/engineering scenario	Candidate quantitative metrics	Suggested testing/data source	Reporting format and validation status	Risk if ignored
				sterilized configuration.		
Branch protection and reintervention feasibility	P1; LBV-specific. The lateral venous angle and medial brachiocephalic–SVC confluence protect different inflows and future catheter, reintervention, and vascular-access resources.	At the lateral venous angle, the left internal jugular and left subclavian veins form the LBV; medially, the left and right brachiocephalic veins form the SVC. These are separate junctions and require separate branch models.	Branch coverage length; residual branch orifice area; branch-flow preservation; feasibility of re-crossing/fenestration; angular matching for Y-shaped or bifurcated configurations.	Branch-model deployment; flow distribution testing; pre-/postoperative three-dimensional reconstruction; guidewire re-crossing tests; fenestrated or bifurcated simulations when needed.	Report branch coverage percentage, flow change, and re-entry success. Avoid unnecessary permanent coverage of important branches whenever possible.	Permanent loss of internal jugular or subclavian resources, limiting future catheters, access creation, and reintervention.
Low foreshortening, deployment precision, and anchoring	P1; LBV-specific. Short landing zones at either anatomical junction convert foreshortening or deployment error directly into branch-coverage risk.	Lateral venous angle and medial brachiocephalic–SVC confluence modeled separately; short landing zones and low tolerance for deployment error.	Axial foreshortening; post-deployment migration; proximal/distal anchoring length; deployment error; oversizing ratio; end-embedding effect.	Transparent anatomical deployment models; patient-specific 3D-printed models; preplanning versus postoperative CTV/venography registration; end-constraint bench testing.	Report nominal length, post-deployment length, anchoring zones, and error range; individualize planning to the relevant lateral or medial junction.	Coverage of contralateral/branch veins, migration, excessive extension into the SVC, and impaired future access or catheter paths.
Axial flexibility, compliance transition, bending fatigue	P1; LBV-specific. Long transverse course, multi-axial angulation, and respiratory motion make fatigue reliability more critical than in straight venous segments. Both landing zones also introduce a stent-to-native-vein stiffness transition.	Dynamic bending from long transverse LBV course, multi-axial angulation, respiratory motion, and upper-limb movement. Possible stiffness mismatch at the proximal and distal stent edges.	Minimum bending radius; bending stiffness; RRF/COF retention after repeated bending; bending fatigue cycles; strut fracture rate. For nitinol constructs, report mean and alternating maximum principal strain at critical locations, together with the compliance profile and edge gradient.	Multi-axial bending fatigue bench; combined respiratory-frequency and upper-limb-motion loading; pre-/post-implant shape matching. Use nonlinear finite-element analysis with nitinol fatigue data obtained under comparable conditions; assess compliance across both stent edges.	Report cycle number, angle, frequency, failure mode, and mechanical decay; use patient imaging to define boundary conditions. Report the critical location, mean and alternating strain, alloy and processing state, surface condition, and test temperature.	Straightening effect, end stress concentration, strut fatigue fracture, local wall injury, and restenosis. An abrupt stent-to-vein stiffness transition may also produce edge deformation or wall-stress concentration.
Hemodynamic optimization	P2; partly LBV-specific. Flow compatibility is a general venous-stent requirement, but LBV curvature, two distinct	The stenotic throat may contain a high-WSS jet, whereas post-repair struts, edge steps, malapposition, or over-straightening may generate low or oscillatory	TAWSS distribution; surface burden outside a prespecified flow-matched reference envelope; OSI; RRT as a derived metric;	Patient-specific virtual deployment; flow-matched smooth-lumen repair and lumen/length/landing-zone-matched reference stent; identical target lumen and	Prespecify a primary disturbed-flow endpoint and comparator margin; report all secondary metrics and full model assumptions. Demonstrate mesh/time-step independence	False acceptance or rejection from comparing non-equivalent pre/post geometries; missed edge recirculation,

Design dimension	Priority and LBV-specific rationale	LBV pathological/engineering scenario	Candidate quantitative metrics	Suggested testing/data source	Reporting format and validation status	Risk if ignored
	junction geometries, multiple inflows, respiratory modulation, and high-flow access return require site-specific adaptation.	shear, steep gradients, persistent recirculation, prolonged residence, pressure loss, or branch-flow redistribution.	spatial WSS gradient; recirculation volume/persistence; reverse-flow fraction; particle washout time; branch-flow fractions; pressure loss.	boundary conditions; flow-loop/PIV or 4D-flow validation; early post-implant flow map with later ISR registration.	and sensitivity to inflow, outlet pressure, respiration, wall compliance, rheology, geometry, and stent representation. No universal LBV threshold is established.	branch compromise, adverse pressure loss, thrombosis, edge stenosis, ISR localization, or patency decay.
Covering durability and active antithrombotic surface	P1 for covering integrity and covering fatigue; P3 for an added active surface. For a covered construct, cover–frame integrity, membrane fatigue, and deformation-induced fabric damage are required P1 components. Baseline thrombogenicity, hemocompatibility, and tissue compatibility are mandatory cross-cutting safety requirements assessed alongside the staged framework, not optional refinements. Only an added active antithrombotic or antiproliferative surface is a P3 option, considered after P1/P2 feasibility.	SG may improve short- to mid-term patency but faces edge stenosis, covering fatigue, thrombosis, and branch-coverage risk in the LBV.	Covering integrity; pinhole/crack formation; leak rate; membrane-scaffold bonding strength; platelet adhesion; clotting time; hemolysis; protein adsorption; edge endothelialization.	Compression-bending fatigue followed by covering inspection; accelerated aging; scanning electron microscopy/microscopy; in vitro hemocompatibility; flow-loop thrombosis testing.	Report membrane integrity and hemocompatibility before and after fatigue; LBV-specific aging and thrombosis thresholds are not established.	Covering failure, intrastent thrombosis, edge stenosis, permanent branch coverage, and reintervention burden.
Local antiproliferative/drug release	P3; exploratory/general. Targets common venous ISR but requires validation after P1 mechanical	Venous stent failure often includes thrombus organization and diffuse intimal thickening; arterial drug-elution experience cannot be copied directly.	Drug load; release half-life; early burst release; tissue-retention concentration; endothelial repair time;	Release kinetics under venous-flow conditions; cell compatibility; tissue penetration model; animal or in vitro venous models; coupling with high-flow,	Report release curve and safety window; no venous/LBV-specific dose, release duration, or efficacy threshold exists.	Delayed healing, local toxicity, hypersensitivity, thrombosis risk, or insufficient antiproliferative

Design dimension	Priority and LBV-specific rationale	LBV pathological/engineering scenario	Candidate quantitative metrics	Suggested testing/data source	Reporting format and validation status	Risk if ignored
	adaptation and branch safety are solved.		smooth muscle proliferation inhibition; inflammatory markers.	low-pressure, and oscillatory shear conditions.		effect causing ISR.
Integrated evaluation and clinical translational endpoints	Translational endpoint. Used to determine whether a P1/P2-screened platform, with or without an added P3 layer, produces net clinical benefit.	A single bench metric cannot predict long-term patency; LBV requires balanced mechanical, hemodynamic, branch, and biological targets.	Technical success; residual stenosis; primary/assisted primary/secondary patency; reintervention interval; branch preservation; stent deformation/fracture; access survival.	Prospective registry; pre-/postoperative imaging follow-up; standardized patency definitions; stratified analysis by device, lesion segment, and prior catheterization.	Report a structured endpoint set; clinical acceptance thresholds for LBV-oriented stent concepts require multicenter prospective studies.	Disconnect between bench performance and clinical outcomes; inability to demonstrate net benefit of a dedicated design.

Supplementary Table S5. P1/P2 validation tests mapped to recognized standards

This table maps the proposed P1/P2 bench and computational tests to recognized consensus standards, and identifies where no current standard fixture reproduces LBV-specific loading. It does not assert regulatory qualification of any device.

Design dimension (priority)	Applicable recognized standard(s)	LBV-specific gap
Uniform radial loading / RRF / COF (P1)	ISO 25539-2:2020 (International Organization for Standardization, 2020); ASTM F3067-26 (ASTM International, 2026) (radial loading); ASTM F2081-06(2022) (ASTM International, 2022) (dimensional attributes)	F2081 addresses dimensions, not functional strength. None defines an LBV acceptance threshold, and the active guide does not define LBV-specific focal loading, amplitudes, or acceptance thresholds.
Focal compression and compression durability (P1)	ASTM F2942-25 (ASTM International, 2025) (separate compression durability guide; also axial, bending, and torsional modes)	F2942-25 does not determine mode relevance, LBV physiological amplitudes or cycle counts, or multi-mode conditions. Custom sternum–aortic loading with patient-derived curvature, respiration, upper-limb motion, and end constraints is required; define diameter reduction unambiguously.
Pulsatile diametric fatigue / durability (P1)	ASTM F2477-24 (ASTM International, 2024)	Pulsatile radial cycling does not reproduce focal extrinsic compression. It addresses only the cardiac-frequency diametric regime, which is one of at least four superimposed regimes over a ten-year implant life; the main text gives order-of-magnitude cycle counts of roughly 4×10^8 (cardiac), of order 10^8 (respiratory), 3.65×10^6 illustrative upper-limb activity events, based on 10^3 events/day over ten years, and about 1.5×10^3 dialysis-session transitions. No standard in this table specifies amplitudes for the latter three, so LBV testing requires superimposed respiratory and upper-limb-motion load spectra derived from dynamic imaging. The upper-limb value is an illustrative activity-event count rather than a measured number of local stent-strain cycles.
Axial / bending / torsion durability (P1)	ASTM F2942-25 (ASTM International, 2025); ASTM F2606-08(2021) (ASTM International, 2021a) (three-point bending)	Boundary conditions should be defined from the CTV-derived curvature and motion envelope. F2942-25 treats modes separately and does not validate an LBV combined-mode spectrum. F2606-08(2021) was developed for three-point bending of balloon-expandable stents and stent systems; it does not directly cover

Design dimension (priority)	Applicable recognized standard(s)	LBV-specific gap
		self-expanding venous stents or stent-grafts and can only inform custom method development.
Kink resistance / flexibility (P1)	ASTM F3505-21 (ASTM International, 2021b)	Single-plane kink testing does not define the LBV curvature-versus-crush trade-off or combined torsion/compression behavior.
Computational fatigue / FEA verification & validation (P1/P2)	ISO 25539-2:2020 (International Organization for Standardization, 2020) (computational annex); ASME V&V 10-2019 (American Society of Mechanical Engineers, 2019) (solid mechanics, P1); ASME V&V 20-2009 (R2021) (American Society of Mechanical Engineers, 2009) (CFD, P2); ASME V&V 40-2018 (American Society of Mechanical Engineers, 2018) (risk-informed medical-device model credibility, P1/P2/FSI)	Standards support FEM/CFD credibility assessment but require an LBV-specific context of use, patient-derived geometry, time-resolved access/subclavian and jugular inflows, SVC/right-atrial outlet conditions, respiratory state, wall and blood models, stent representation, experimental validation, and risk-based sensitivity/uncertainty analysis. No model or threshold has been validated for LBV stenting.
Covering / fabric durability (P1, required for covered constructs)	ISO 25539-1:2017 (International Organization for Standardization, 2017) (endovascular prostheses); ASTM F2942-25 (ASTM International, 2025) (stent-graft non-radial durability); ISO 7198:2016 (International Organization for Standardization, 2016) only for relevant graft-material method development	ISO 7198:2016 excludes catheter-delivered endovascular prostheses and vascular stents; it cannot serve as the governing stent-graft standard. ISO 25539-1 and F2942-25 provide broader frameworks but do not define LBV covering-fatigue, interface, aging, hemocompatibility, or branch-safety conditions.

Scope note. ASTM F3067-26 (ASTM International, 2026) is radial loading—not focal LBV crush; ASTM F2942-25 (ASTM International, 2025) supplies separate non-radial durability modes but no LBV boundary conditions; and ISO 7198:2016 (International Organization for Standardization, 2016) is limited graft-material guidance rather than the governing standard for endovascular stent-grafts.

Supplementary Table S6. Claim–evidence matrix for key biomechanical and mechanistic propositions

This table maps evidence to individual key claims rather than assigning a permanent category to each paper. A single source may contribute to several claims, and one claim may require more than one evidence layer. Evidence proximity indicates anatomical and experimental closeness to the specific LBV inference, not methodological quality. The permissible-interpretation and required-validation columns make explicit what the cited evidence can support and where an LBV-specific gap remains. Proximity entries name the applicable tiers of the four-tier scheme defined in the main text and are joined with a plus sign when a claim rests on more than one tier. The final row records the staged P1/P2/P3 structure itself, which is a proposed organizing scheme rather than an evidence claim and therefore sits outside the four-tier scheme. Rows carrying the two hypotheses stated in the main text are flagged H1 and H2.

Key claim	Closest supporting evidence	Evidence proximity	Permissible interpretation in this Review	LBV-specific validation required
The retrosternal LBV contains a focal sternum–aortic compression window.	LBV imaging/anatomy and HD cohorts: Itkin et al., 2004; Shi et al., 2014; Vertemati et al., 2020; Wan et al., 2024.	Direct LBV	Focal external compression is a credible site-specific boundary condition. Its reported frequency refers largely to any detectable narrowing rather than to high-grade disease: in the largest cited HD series, compression was mild in most affected patients and severe in only a small minority, so prevalence figures must be read with the severity distribution attached.	Dynamic CTV/venography with segment-resolved deformation and stratification by catheter history and access flow.

Key claim	Closest supporting evidence	Evidence proximity	Permissible interpretation in this Review	LBV-specific validation required
Compression geometry exists in the general adult population independently of dialysis.	Non-dialysis venographic cohort: Guo et al., 2017 (212 asymptomatic adults; left innominate narrowing >25% area loss at the crossing point in 35.4%, with a smaller aortic arch–to–sternum space and a more proximal crossing site as independent correlates).	Direct LBV (non-HD population)	The compression window is an anatomical baseline rather than a dialysis-induced lesion, which supports the unmasking framing used in this Review. It does not establish that compression causes HD-related stenosis, and the cohort was asymptomatic and non-dialysis.	HD and non-HD cohorts measured with one imaging protocol and one narrowing definition, reporting corridor width, crossing-point position, and severity distribution, with access side and flow recorded.
The reported corridor geometry implies compression beyond the range covered by published venous-stent bench characterization.	LBV corridor measurement plus venous-stent bench comparison: Shi et al., 2014 (sternum-aortic space 4.55 ± 2.67 mm with stenosis versus 15.25 ± 6.12 mm without); Dabir et al., 2018 (devices characterized only down to 50% of nominal diameter).	Direct LBV (anatomy) + Analogous engineering	An arithmetic sizing of the plausible compression range: a 12 mm device constrained within a 4.6 mm corridor sits near 38% of nominal diameter, below the lowest bench-characterized level. This is not a design threshold. The corridor was measured statically, without an implanted device, in a 9-versus-10-patient cohort, and corridor width and residual lumen are not independent measurements in an already narrowed vein.	In-situ corridor measurement with a device in place; the width of the sternum-aortic contact footprint, which sets the loaded arc and is unreported; and bench characterization extended below 50% of nominal diameter.
The LBV warrants separate treatment from the right brachiocephalic vein.	Comparative anatomy and caliber studies: Vertemati et al., 2020 (longer, more angulated course); White and Deeb, 2023 (sternum–aortic crossing not shared by the right brachiocephalic vein); Agarwal, 2015 (smaller baseline left internal jugular caliber in roughly one-third of patients).	Direct LBV	Convergent anatomical asymmetry justifies analyzing the LBV as a distinct segment. No head-to-head comparison of left- versus right-sided stent performance or lesion incidence supports a quantitative side effect.	Side-stratified cohorts using identical imaging and patency definitions, reporting lesion incidence, geometry, device deformation, and reintervention separately for the two brachiocephalic veins.
H1 (mechanical). Localized unidirectional crush and uniform circumferential radial loading are not interchangeable.	Venous-stent bench comparisons and mechanics reviews: Dabir et al., 2018; Schwein et al., 2018; Lim and Black, 2022.	Analogous engineering + Direct LBV (anatomy)	Focal crush and circumferential radial loading are distinct and complementary modes. Neither a high RRF nor a single crush value establishes overall mechanical superiority.	Matched device size, length, oversizing, deployment state, temperature, curvature, end constraints, and fixtures; defined diameter reduction; prespecified comparator panel, safety criteria, endpoint-specific margins, confidence intervals, and trade-off reporting.
Recovery after unloading is a mechanical attribute distinct from peak crush resistance.	Venous-stent compression and deformation testing: Dabir et al., 2018; Schwein et al., 2018; Maleckis et al., 2017, 2018; Lim and Black, 2022.	Analogous engineering	A device may resist a given compression load yet recover incompletely once the load is removed, so peak crush load alone does not describe behavior under the repeated compression-release cycling implied by respiration and limb motion. No acceptable recovery fraction is defined.	Paired crush and post-unloading recovery measurement at defined residual nominal diameters, with hold duration, cycle count, temperature, and end constraints reported alongside the peak load.
LBV curvature and motion	LBV geometry plus non-LBV	Direct LBV +	Bending, torsion, axial motion, and	Dynamic imaging and activity-derived

Key claim	Closest supporting evidence	Evidence proximity	Permissible interpretation in this Review	LBV-specific validation required
justify multi-mode deformation and fatigue assessment.	deformation studies: Vertemati et al., 2020; Cheng et al., 2020; Maleckis et al., 2017, 2018.	Analogous engineering	compression are candidate coupled loads; no LBV load spectrum is established. For nitinol constructs, cycle count alone does not define fatigue safety.	load spectra followed by combined-mode fatigue testing in LBV geometry. For nitinol constructs, report the FE-derived mean and alternating components of maximum principal strain at critical locations and compare them with a material- and processing-matched strain-based constant-life diagram.
Stent-to-native-vein compliance mismatch may concentrate deformation and wall stress at the landing zones.	Arterial compliance-matching stent mechanics: Berry et al., 2002.	Analogous engineering.	A candidate solid-mechanics mechanism distinct from the WSS/OSI/RRT pathway; not evidence that compliance mismatch causes LBV edge hyperplasia.	Axial compliance or incremental-stiffness profiling across both stent edges, with local wall strain/stress assessment and correlation with later edge outcomes.
High-flow access may amplify, but does not by itself establish, LBV stenosis or stent failure.	AVF/AVG hemodynamic and venous-flow studies: Ene-Iordache and Remuzzi, 2012; Franzoni et al., 2016; Ninno et al., 2025.	Adjacent HD-CVS + Mechanistic hypothesis	High flow is treated as an interacting stressor or unmasking factor, not a universal independent cause.	Flow-stratified LBV cohorts with pre/post-access imaging, pressure/flow measurement, and longitudinal lesion localization.
H2 (hemodynamic). Stent edges, struts, curvature, lateral-venous-angle geometry, medial brachiocephalic–SVC geometry, and access-flow conditions may create non-physiologic shear, recirculation, and prolonged residence.	AVF/AVG, venous-stent, and boundary-condition CFD studies: Ene-Iordache and Remuzzi, 2012; Franzoni et al., 2016; McCarthy et al., 2025; Torikai et al., 2026; Zhang et al., 2026.	Analogous engineering + Mechanistic hypothesis	Low or oscillatory shear, high WSS gradients, recirculation, residence, branch flow, and pressure loss are candidate P2 metrics. Neither a universal LBV threshold nor a monotonic causal direction is established.	Flow-matched smooth-lumen and reference-stent comparators; early post-implant flow mapped to later ISR; time-resolved inflow/outlet data; respiratory, wall, rheology, geometry, mesh, and strut-representation sensitivity and uncertainty analyses.
ISR and patency decay are documented, but their LBV tissue composition and causal sequence are unresolved.	HD-CVS/stent outcomes and non-LBV pathology: Liu et al., 2024; Yu et al., 2024; Chawla et al., 2025; Williams et al., 2023.	Adjacent HD-CVS + Mechanistic hypothesis (tissue composition only)	Clinical lumen loss can be stated; thrombus organization and neointimal hyperplasia must not be assumed equivalent or obligatory steps.	LBV stent-restenosis tissue, histomorphometry, thrombus-age markers, spatial sampling, and imaging–pathology correlation.
Deployment near either the lateral venous angle or medial brachiocephalic–SVC confluence creates junction-specific branch-preservation, foreshortening, and re-entry constraints.	Central venous guidance/anatomy and branch-preservation experience: Lok et al., 2020; Ginsburg et al., 2015; LBV Y-shaped stenting case, Theodoridis et al., 2024; Hu et al., 2024.	Direct LBV case + Adjacent HD-CVS + Analogous engineering	These are candidate design constraints; no LBV-specific acceptable coverage or deployment-accuracy threshold is established.	Anatomical branch models, deployment simulations, branch-flow testing, and prospective reporting of coverage and re-entry outcomes.
Piezo1 and ROS/NF-κB may link mechanical stimuli to remodeling.	Cellular and general vascular mechanobiology: Helmcke et al., 2009; Chandel et al., 2000; Reynaert et al., 2006; Shinge et al., 2021, 2022;	Mechanistic hypothesis	These pathways identify exploratory biomarkers and experiments; they do not establish a causal LBV ISR pathway or therapeutic target.	LBV tissue, venous dynamic-compression models, spatial omics, and imaging–CFD/FSI–pathology correlation.

Key claim	Closest supporting evidence	Evidence proximity	Permissible interpretation in this Review	LBV-specific validation required
	Zeng et al., 2025.			
The staged P1/P2/P3 structure organizes the evaluation of an LBV-oriented stent concept.	No direct evidence. The structure is derived from the gaps recorded in the rows above and from the standards mapping in Supplementary Table S5.	Framework proposal; outside the four-tier evidence scheme	The staging is an organizing and reporting scheme for evidence generation. It is not a regulatory pathway, has not been validated, and passing P1 and P2 is not shown to predict clinical outcome; P3 does not make baseline safety attributes optional.	Prospective demonstration in an LBV imaging–patency registry that prespecified P1 and P2 outputs correlate with residual stenosis, stent deformation, patency, and reintervention.

Note. For ISR, documented lumen loss and patency decay are separated from their unverified tissue composition. Thrombus organization and neointimal hyperplasia can interact but must not be assumed to be equivalent processes or obligatory consecutive steps without LBV tissue evidence.

Supplementary Table S7. Full stage-gate logic for the P1/P2/P3 validation roadmap

This table preserves the complete entry criteria, comparator strategy, endpoint-specific decision rules, and failure handling formerly presented as main-text Table 6. P1 and P2 are required base-platform gates; P3 is optional only for an added drug, active coating, or functional surface. Baseline thrombogenicity, hemocompatibility, and tissue compatibility remain mandatory cross-cutting safety requirements. It is a proposed research framework, not a regulatory qualification threshold, clinical algorithm, device ranking, or evidence of efficacy.

Stage	Entry criteria (required inputs)	Core test and comparator	Gate criterion to advance	Failure handling
P1: mechanical / deployment	Patient-derived CTV geometry and load envelope; finalized candidate configuration; prespecified indication-relevant comparator panel or matched internal prototypes; locked diameter, length, oversizing, deployment state, fixture, temperature, curvature, end constraints, endpoints, margins, sample-size rationale, and analysis plan.	Focal compression at 10%, 25%, 50% and 65% diameter reduction (90%, 75%, 50% and 35% residual nominal diameter), with imaging-derived primary range; chronic compression/recovery and multi-axial fatigue; for nitinol constructs, nonlinear FE assessment of the mean and alternating components of maximum principal strain at critical locations against matched constant-life data; axial compliance or incremental-stiffness profiles and edge gradients; RRF/COF, bending, foreshortening, deployment, branch-access, and re-entry tests. Candidate and comparators are tested side by side in final sterilized configurations under identical conditions. ASTM F2942-25 (ASTM International, 2025)	All prespecified absolute safety criteria met (including no fracture, loss of integrity, deployment failure, or irreversible branch obstruction). For each continuous endpoint, the 95% CI satisfies its justified superiority/equivalence/non-inferiority margin. Full trade-offs are reported; no safety-critical failure is offset by strength elsewhere. Pareto analysis may summarize non-dominated profiles but is not an acceptance rule by itself.	Identify the failed endpoint and redesign the relevant segment, material, deployment system, or boundary interaction. Repeat the locked comparison; do not change comparator or margin after observing results. A candidate that fails a safety-critical P1 criterion does not proceed to P2.

Stage	Entry criteria (required inputs)	Core test and comparator	Gate criterion to advance	Failure handling
		informs separate compression durability but does not define LBV loading or multi-mode conditions.		
P2: hemodynamic	P1 gate passed; patient-specific geometry and finalized target lumen/landing zones; time-resolved access/subclavian and internal-jugular inflows; SVC/right-atrial outlet pressure or impedance; prespecified respiratory state, wall model, blood rheology, stent representation, and uncertainty ranges.	Candidate virtual deployment compared with (1) a flow-matched patient-specific smooth-lumen repair and (2) a diameter-, length-, landing-zone-, and lumen-gain-matched reference stent under identical conditions. The diseased pre-intervention model is contextual only. CFD/FSI is supported by flow-loop/PIV or 4D-flow validation where feasible.	Non-inferior or improved prespecified disturbed-flow burden versus the reference stent; preserved branch-flow distribution; no new persistent edge recirculation, adverse pressure loss, or high-gradient jet; conclusions robust to sensitivity and uncertainty analyses. Advancement reflects movement toward a flow-matched reference envelope—not maximum WSS or mandatory improvement over the stenotic baseline.	Revise mesh, edge transition, diameter, apposition, landing zone, or deployment geometry and repeat P2. A biological or surface layer cannot compensate for an unfavorable base-platform flow field.
P3 (optional): added biological or surface layer	P1 and P2 gates passed on the same platform, with mandatory cross-cutting safety requirements met.	Venous-flow release kinetics; platelet, coagulation, and complement assays; endothelial and smooth-muscle-cell compatibility; coating integrity after crimping, deployment, bending, and compression, compared against the uncoated P1/P2-screened platform as the reference baseline.	Added layer does not degrade the mechanical or hemodynamic performance of the P1/P2-screened platform and shows acceptable incremental hemocompatibility and coating integrity in ex vivo or animal flow-loop testing; any change that alters platform mechanics or the flow field triggers repeat P1 or P2 evaluation of the affected domain.	Revert to the uncoated P1/P2-screened platform; treat the added biological or surface layer as a separate future iteration rather than a blocker to translational progress.
Translational endpoint	P1 and P2 gates passed on the same platform, with mandatory cross-cutting safety requirements met. If an added drug, active coating, or other functional surface is included, that configuration must also pass Gate 3; a P1/P2-screened uncoated platform may proceed without an added P3 layer.	Prospective imaging-patency registry with standardized lesion definitions; compared against current PTA/BMS/SG boundary evidence summarized in Table 1.	Primary/assisted/secondary patency and access-survival outcomes at defined follow-up exceed the current PTA/BMS/SG boundary evidence. No numeric target is prespecified by this review.	Return the platform to P1, P2, or P3 iteration as appropriate; absence of clinical advantage over current boundary evidence is itself an informative outcome, not a terminal failure of the framework.

Supplementary note

Mechanistic and device information in Supplementary Box S1, Supplementary Table S6, and Supplementary Tables S2 and S5 should be interpreted according to evidence proximity. These details support candidate testing dimensions and design hypotheses for LBV-oriented stent concepts, but they do not demonstrate definitive superiority of any commercial device in LBV patients. Supplementary Table S2 documents the search approach and evidence-use boundaries; it is not a systematic-review selection log. Terminology and formatting were

standardized before submission: p values are italicized and written as $p < 0.05$; wall shear stress is reported in SI units (Pa; 1 Pa = 10 dyne/cm^2); residual nominal diameter, rather than compression ratio, is used for the degree of imposed narrowing; and landing zone is used consistently.
